# CD11c^hi^ Dendritic Cells Regulate Ly-6C^hi^ Monocyte Differentiation to Preserve Immune-privileged CNS in Lethal Neuroinflammation

**DOI:** 10.1038/srep17548

**Published:** 2015-12-02

**Authors:** Jin Hyoung Kim, Jin Young Choi, Seong Bum Kim, Erdenebelig Uyangaa, Ajit Mahadev Patil, Young Woo Han, Sang-Youel Park, John Hwa Lee, Koanhoi Kim, Seong Kug Eo

**Affiliations:** 1 College of Veterinary Medicine and Bio-Safety Research Institute, Chonbuk National University, Iksan 54596, Republic of Korea; 2 Department of Bioactive Material Sciences, Graduate School, Chonbuk National University, Jeonju 54896, Republic of Korea; 3 Department of Pharmacology, Pusan National University, School of Medicine, Yangsan 50612, Republic of Korea

## Abstract

Although the roles of dendritic cells (DCs) in adaptive defense have been defined well, the contribution of DCs to T cell-independent innate defense and subsequent neuroimmunopathology in immune-privileged CNS upon infection with neurotropic viruses has not been completely defined. Notably, DC roles in regulating innate CD11b^+^Ly-6C^hi^ monocyte functions during neuroinflammation have not yet been addressed. Using selective ablation of CD11c^hi^PDCA-1^int/lo^ DCs without alteration in CD11c^int^PDCA-1^hi^ plasmacytoid DC number, we found that CD11c^hi^ DCs are essential to control neuroinflammation caused by infection with neurotropic Japanese encephalitis virus, through early and increased infiltration of CD11b^+^Ly-6C^hi^ monocytes and higher expression of CC chemokines. More interestingly, selective CD11c^hi^ DC ablation provided altered differentiation and function of infiltrated CD11b^+^Ly-6C^hi^ monocytes in the CNS through Flt3-L and GM-CSF, which was closely associated with severely enhanced neuroinflammation. Furthermore, CD11b^+^Ly-6C^hi^ monocytes generated in CD11c^hi^ DC-ablated environment had a deleterious rather than protective role during neuroinflammation, and were more quickly recruited into inflamed CNS, depending on CCR2, thereby exacerbating neuroinflammation *via* enhanced supply of virus from the periphery. Therefore, our data demonstrate that CD11c^hi^ DCs provide a critical and unexpected role to preserve the immune-privileged CNS in lethal neuroinflammation *via* regulating the differentiation, function, and trafficking of CD11b^+^Ly-6C^hi^ monocytes.

The central nervous system (CNS) is considered to be immune privilege tissue in which adaptive and innate immune responses are highly controlled. CNS immune privilege is based on multiple factors, including its isolation from peripheral immune system by the blood-brain barrier (BBB), lack of draining lymphatics, and the apparent immunocompetence of microglia^1^. However, the concept of CNS immune privilege has seemingly become swollen and imprecise by the apparent fact that the CNS is neither isolated nor passive in its interactions with the peripheral immune system; peripheral immune cells can cross the intact BBB^2^, and CNS neurons and glia actively regulate the infiltrated macrophage and lymphocyte response^2^. Moreover, recent discovery of the CNS lymphatic system indicates that the CNS actively communicates with peripheral immune systems^3^. CNS infiltration by peripheral innate immune cells is critical for protective host defense against infection and for repair after stroke or physical trauma^2^. However, restraint of CNS infiltration is also required because hematogenous inflammation causes profound damage if the reaction is excessive or inappropriate. Therefore, peripheral innate immune cells are considered to be key players in maintaining functional homeostasis of the CNS under steady and/or neuroinflammatory conditions. However, in-depth evidence for the role of peripheral innate immune cells in maintaining CNS immune privilege needs to be further accumulated.

CNS infiltration of CD11b^+^Ly-6C^hi^ monocytes is a hallmark of CNS inflammation, including neurotropic viral infection^4^. These cells migrate into the infected brain, where they differentiate into DCs, macrophages, and arguably microglia population^4^,^5^,^6^. However, a debatable role of CD11b^+^Ly-6C^hi^ monocytes is their potential contribution to immunopathology within the immune-privileged CNS. In several models of CNS disease, CD11b^+^Ly-6C^hi^ monocytes cause significant damage and destruction to the immune-privileged CNS, directly contributing to morbidity and mortality^5^,^6^,^7^,^8^. In contrast, CNS infiltration by leukocytes, including CD11b^+^Ly-6C^hi^ monocytes, supports their protective role during CNS inflammation^9^,^10^,^11^,^12^, which suggests that CD11b^+^Ly-6C^hi^ monocytes may be beneficial. Therefore, the precise differentiation pathways and functions of CD11b^+^Ly-6C^hi^ monocytes in the inflamed CNS remains a contentious issue, and the contributions of monocyte-derived subsets to clearance of neurotropic virus and immunopathology within the immune-privileged CNS are not well-defined.

Recently, a detailed map of the relationship between monocytes and dendritic cells (DCs) and their progenitors (CD115^+^CX_3_CR1^+^ monocyte-macrophage DC precursor [MDP]) has begun to be uncovered^13^,^14^. The mononuclear phagocyte system represents a subpopulation of leukocytes originally described as a population of bone marrow (BM)-derived myeloid cells that circulate in the blood as monocytes, and subsequently differentiate into tissue macrophages, which could be also derived from hematopoietic stem cell (HSC)-independent embryonic progenitors^14^. DCs are also derived from a distinct lineage of mononuclear phagocytes (common DC progenitor [CDP] derived from the CD115^+^CX_3_CR1^+^ MDP), and then specialized into antigen presentation for initiating immune responses^15^. In addition, monocytes and macrophages are recently recognized to be renewed independently of DCs from a committed progenitor called common monocyte progenitors (CD115^+^CD135^−^Ly-6C^+^CD11b^−^ cMoP) derived from CD115^+^CD135^+^Ly-6C^−^CD11b^−^ MDP^13^,^14^. Murine blood monocytes can be further subpopulated by the expression of Ly-6C and CX_3_CR1 into Ly-6C^hi^CX_3_CR1^lo^CCR2^+^CD62L^+^ and Ly-6C^lo^CX_3_CR1^hi^CCR2^−^CD62L^−^ monocytes^16^. Whereas CD11b^+^Ly-6C^lo^ monocyte subset is recruited to normal tissue and develops into resident M2 macrophages that function in host defense and repair after tissue injury^16^,^17^, CD11b^+^Ly-6C^hi^ monocyte subset is specifically recruited to inflammatory sites in various inflammation conditions by CCL2 (known as MCP-1), and these cells become classically activated M1 macrophages and/or Tip-DCs^16^,^17^. Although the role of DCs in adaptive host defense by instructing CD4^+^ and CD8^+^ T cells is well established, the potential contribution of peripheral DCs to T cell-independent innate host defense and to subsequent immunopathology in specialized tissues, such as immune-privileged CNS, is poorly understood. In particular, the role of DCs in regulating the functions of innate immune CD11b^+^Ly-6C^hi^ monocytes during neuroinflammatory progression in immune-privileged CNS have not yet been addressed in depth.

Therefore, the aims of this study were to investigate i) the potential contribution of peripheral DCs to the differentiation and function of CD11b^+^Ly-6C^hi^ monocytes, as well as ii) the deleterious or beneficial roles of infiltrated CD11b^+^Ly-6C^hi^ monocytes in the progression of neuroinflammation within the immune-privileged CNS, using Japanese encephalitis (JE) model which is lethal neuroinflammation caused by JE virus (JEV). JEV is considered to be the most prevalent cause of viral encephalitis with approximately 67,900 cases reported annually^18^. Of these cases, about 25–30% are fatal and 50% result in permanent neuropsychiatric sequelae, for which JE is considered to be more fatal than West Nile (WN) encephalitis resulting in a fatality of 3–5% (1,100 deaths/29,000 symptomatic infection)^19^. In order to address the suggested aims in JE model, we used an inducible mouse model allowing selective ablation of CD11c^hi^PDCA-1^int/lo^ DCs, but not CD11c^int^PDCA-1^hi^ pDCs. Our data demonstrate that selective ablation of CD11c^hi^ DCs provided altered differentiation of infiltrated CD11b^+^Ly-6C^hi^ monocytes in the CNS through Flt3-L and GM-CSF, which was closely associated with severely enhanced JE. Furthermore, CD11b^+^Ly-6C^hi^ monocytes generated in CD11c^hi^ DC-ablated environment were found to be more quickly recruited into the inflamed CNS in a CCR2-dependent manner, thereby accelerating the severity of neuroinflammation within the CNS. Therefore, our results illuminate an unrecognized role of CD11c^hi^ DCs in maintaining the functional homeostasis of infiltrated CD11b^+^Ly-6C^hi^ monocytes in the immune-privileged CNS, thereby affecting the control of acute neurological diseases.

## Results

### Selective ablation of CD11c^hi^PDCA-1^int/lo^ DCs, but not CD11c^int^PDCA-1^hi^ pDCs, exacerbates JE

CD11c-DTR transgenic mice, which express diphtheria toxin receptor (DTR) gene under control of a cloned *Itgax* promoter and thus allow conditional DC depletion upon DT injection, has been a milestone in DC research^20^,^21^. However, along with contrasting results in the strength of DC subset depletion^20^,^21^,^22^,^23^,^24^,^25^, it was recently reported that CD11c-DTR mice showed side effect such as systemic neutrophilia after DT injection to deplete CD11c^+^ DCs^22^,^23^. Because the majority of published literatures used 8–11 ng/g DT to deplete CD11c^+^ DCs^20^,^21^,^22^,^23^,^24^,^25^, the injection doses of DT were carefully titrated to achieve selective depletion of CD11c^hi^ DCs. While the injection of low DT dose (2 ng/g) provided selective depletion of CD11c^hi^PDCA-1^int/lo^ DCs with slightly increased CD11c^int^PDCA-1^hi^ pDC frequency and number, the injection of higher DT dose (10 and 25 ng/g) depleted CD11c^hi^PDCA-1^int/lo^ DCs as well as CD11c^int^PDCA-1^hi^ pDCs (Fig. S1A-B). To better understand the depletion of CD11c^hi^ DCs, we analyzed the frequency and total number of DC subsets (CD11c^hi^CD8α^+^, CD11c^hi^CD11b^+^, CD11c^hi^PDCA-1^int/lo^, and CD11c^int^PDCA-1^hi^ pDC) after DT injection (2 ng/g). Our data revealed that subpopulations of CD11c^hi^ DCs, but not CD11c^int^PDCA-1^hi^ pDCs, were selectively depleted by DT (2 ng/g) injection (Fig. S1C-D). Also, the major populations of other lymphoid and myeloid cells in spleen, blood, and bone marrow (BM), including B (CD3^−^CD19^+^), NK (CD3^−^NK1.1^+^) cells, and CD11b^+^Ly-6C^hi^ monocytes, were not affected by DT (2 ng/g) injection (Fig. S2A–C). Furthermore, a repetitive systemic injection of such titrated DT (2 ng/g) did not cause systemic neutrophilia^22^,^23^ as well as any apparent changes in body weight, signs of illness, or long-term defects during monitoring periods that ranged from 7 to 10 days (Fig. S3A–C).

**Figure 1:**
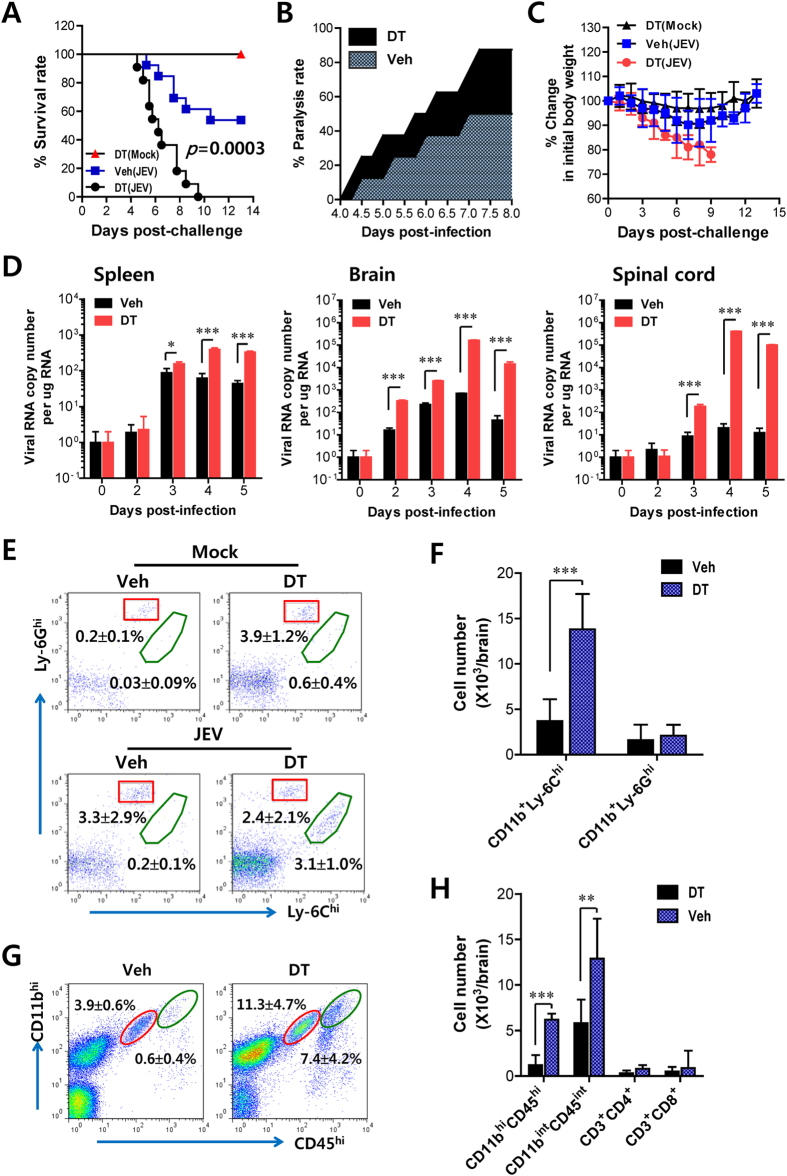
Ablation of CD11c^hi^PDCA-1^int/lo^ DCs, but not CD11c^int^PDCA-1^hi^ pDCs, exacerbates neuroinflammation. CD11c-DTR mice were injected i.p. with DT every other day from -1 to 7 days after JEV infection (1.5 × 10^7^ pfu/mouse). (**A**) Survival rate. The proportion of surviving mice per group (*n* = 11–13) was monitored until day 13 after infection. (**B**) The proportion of mice showing paralysis. The proportion of JEV-infected CD11c-DTR mice showing paralysis was determined every 6 h from 4 to 8 dpi. (**C**) Changes in body weight. Data is expressed as the average percentage ± SD of body weight relative to the time of challenge. (**D**) Viral burden. Viral burden in the spleen, brain, and spinal cord of mice infected with JEV was assessed by real-time qRT-PCR at the indicated dpi. (**E**,**F**) Early infiltration of inflammatory CD11b^+^Ly-6C^hi^ monocytes. After vigorous heart perfusion on the 3rd dpi, the frequency (**E**) and total number (**F**) of CD11b^+^Ly-6C^hi^ and CD11b^+^Ly-6G^hi^ cells infiltrated into the brain were analyzed by flow cytometric analysis. The values in representative dot-plots show the average percentage ± SD of Ly-6C^hi^ and Ly-6G^hi^ cells after gated on CD11b^+^ cells. (**G**,**H**) Analysis of CNS microglia. The frequency (**G**) and total number (**H**) of CD11b^int^CD45^int^ (resting microglia) and CD11b^hi^CD45^hi^ (activated microglia and macrophages) were determined on the 3rd dpi. Data in dot-plots and bar graphs denote the average percentage/number ± SD of the indicated cell population derived from at least three individual experiments (*n* = 4–7). *p < 0.05; **p < 0.01; ***p < 0.001 compared with the levels of the indicated group.

Using this selective depletion model of CD11c^hi^ DCs, we evaluated the impact of CD11c^hi^ DCs on JE progression (Fig. 1A). CD11c^hi^ DC-depleted mice succumbed to JE with 100% mortality, whereas vehicle-treated CD11c-DTR mice showed around 50% mortality. Also, CD11c^hi^ DC-depleted mice showed more rapid development of neurological disorder (paralysis) started from 3–4 dpi by up to 100% rate (Fig. 1B), and severely reduced body weight (Fig. 1C), as compared to vehicle-treated CD11c-DTR mice. Although any side effects such as neutrophilia, changes of body weight, and signs of illness were not shown by DT (2 ng/g) injection, we also used B6 recipients reconstituted by BM cells derived from CD11c-DTR mice to rule out residual side effect^24^,^25^. Consistently, CD11c-DTR BM recipients showed 100% mortality, faster neurological signs, and marked reduction of body weight following depletion of CD11c^hi^PDCA-1^int/lo^ DCs by DT (2 ng/g) injection (Fig. S4A–C). Additionally, we determined viral RNA levels in lymphoid (spleen) and inflammatory CNS tissues (brain and spinal cord) (Fig. 1D). CD11c^hi^ DC ablation provided increased viral burden in lymphoid and CNS tissues. Of note, the inflammatory CNS tissues (brain and spinal cord) of CD11c^hi^ DC-depleted mice contained viral burden with 100- to 1,000-fold increased levels 4 and 5 dpi, but not 2 and 3 dpi. These results indicate that selective ablation of CD11c^hi^ DCs provides enhanced susceptibility to JE and facilitates the increase of viral burden in CNS tissues.

To further characterize JE in CD11c^hi^ DC-ablated mice, we examined infiltrated myeloid-derived cell populations (Ly-6C^hi^ monocyte and Ly-6G^hi^ granulocyte) in the CNS at 3 dpi, a time point before showing neurological disorder. The infiltrated populations of granulocytes (CD11b^+^Ly-6G^hi^) were comparable between vehicle- and DT-treated CD11c-DTR mice in frequency and absolute number following JEV infection (Fig. 1E,F). In contrast, CD11b^+^Ly-6C^hi^ monocytes were prominently infiltrated in the CNS of DC-ablated mice with a 10-fold increase over vehicle-treated mice. Notably, the absolute number of CD11b^+^Ly-6C^hi^ monocytes increased 3- to 4-fold in DC-ablated mice. Furthermore, based on the CNS myeloid cell classification of Ford *et al.*^26^, we enumerated resting microglia (CD11b^int^CD45^int^) and activated microglia/macrophages (CD11b^hi^CD45^hi^) in the CNS at 3 dpi. The proportions (Fig. 1G) and absolute numbers (Fig. 1H) of resting microglia and activated microglia/macrophages were increased in CD11c^hi^ DC-depleted mice. Of note, activated microglia/macrophages (CD11b^hi^CD45^hi^) were detected at a 10-fold increased level in CD11c^hi^PDCA-1^int/lo^ DC-depleted mice. Also, CD11b^+^CD45^−^ leukocytes containing many Ly-6C^+^Ly-6G^−^F4/80^int^ cells were detected in the brain of CD11c^hi^ DC-ablated mice with increased levels, compared to vehicle-treated CD11c-DTR mice.

**Figure 2:**
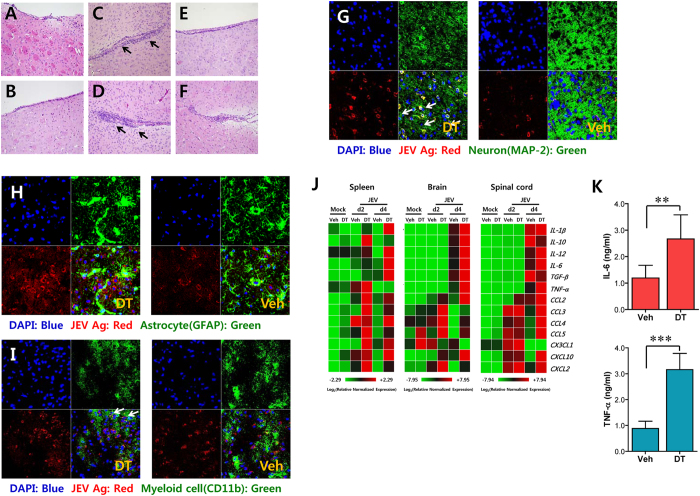
Histological/confocal examinations and early CC chemokine expression in the CNS of DC-ablated mice during lethal neuroinflammation. (**A**–**F**) Representative photomicrographs of brain sections stained with H&E. Photomicrographs were taken from coronal sections of the septo-striatal regions of brain in vehicle (**A**) and DT (**B**)-treated CD11c-DTR mice, DT-treated CD11c-DTR mice followed by JEV infection ((**C**) 4 dpi, (**D**) 6 dpi), and vehicle-treated CD11c-DTR followed by JEV infection ((**E**) 4 dpi, (**F**) 6 dpi). The arrows denote the area of interest. (**G**–**I**) Confocal microscopic examination of brain sections. Brain sections prepared at 4 dpi were co-stained for JEV antigen (red), the nuclear stain DAPI (blue), and the neuronal marker MAP-2 (green) (**G**), the astrocyte-specific marker GFAP (green) (**H**), or the microglial/macrophage cell-marker CD11b (green) (**I**). White arrows indicate double-positive cells. Data are representative of sections from at least four mice per treated group. (**J**) Heatmap showing the expression of cytokines and chemokines in spleen, brain, and spinal cord. The expression level of each cytokine and chemokine was normalized to β-actin and is displayed as the average of at least four independent samples, according to the indicated color on a log_2_ scale. (**K**) Systemic IL-6 and TNF-α levels. The levels of serum IL-6 and TNF-α in sera were determined by conventional ELISA at 3 dpi. Data in bar graphs denote the average ± SD of cytokine levels derived from three individual experiments (*n* = 4–5). *p < 0.05; **p < 0.01; ***p < 0.001 compared with the levels of the indicated group.

To better understand a severe neuroinflammatory reaction in the immune-privileged CNS of CD11c^hi^PDCA-1^int/lo^ DC-depleted mice, we examined the cellular distribution of JEV antigen in the brain by histological examinations and confocal microscopy using antibodies against JEV NS1/E proteins and specific CNS cell types. Significantly, CD11c^hi^PDCA-1^int/lo^ DC-depleted mice had early recruitment of inflammatory cells into the brain, compared to vehicle-treated CD11c-DTR mice after JEV infection (Fig. 2A–F). The majority of infected cells were observed to be neuron cells, since prominent costaining was observed between neuronal marker MAP-2 and the JEV antigen (Fig. 2G). Of note, a higher frequency of JEV antigen was detected in the brains of DC-ablated mice. In contrast, no costaining was detected between GFAP^+^ astrocytes and JEV antigen, indicating that astrocytes were not infected with JEV (Fig. 2H). Finally, a small subset of CD11b^+^ myeloid cells costained positively with JEV antigen, and enhanced frequencies of JEV-infected CD11b^+^ cells were observed in CD11c^hi^PDCA-1^int/lo^ DC-depleted mice (Fig. 2I). Taken together, these results indicate that CD11c^hi^ DCs are essential for the control of neuroinflammatory progression in immune-privileged CNS following JEV infection.

Because neuroinflammation caused by neurotropic viruses is indirectly derived from CNS degeneration caused by robust immunological responses, such as the uncontrolled secretion of cytokines and chemokines and the resultant activation of microglia and astrocytes^27^,^28^,^29^, knowledge of the expression levels of cytokines and chemokines within the CNS can be required for further explanation of neuroinflammation. Therefore, we examined the expression of cytokines and chemokines in inflammatory tissues. We found that the ablation of CD11c^hi^PDCA-1^int/lo^ DCs provided greatly enhanced expression levels of cytokines and chemokines in the spleen and in the CNS, including brain and spinal cord (Fig. 2J). Of note, increases in the expression levels of CC chemokines (CCL2, CCL3, CCL4, and CCL5) in the immune-privileged CNS of DC-ablated mice preceded the increases in expression of pro-inflammatory cytokines and CXC chemokines, suggesting that early expression of CC chemokines may induce infiltration of leukocytes into the CNS of DC-ablated mice. We also measured the levels of systemic IL-6 and TNF-α in the serum of JEV-infected mice at 3 dpi. Trends toward increased levels of IL-6 and TNF-α were observed in the serum of CD11c^hi^PDCA-1^int/lo^ DC-depleted mice (Fig. 2K). Collectively, these results indicate that CD11c^hi^ DC ablation exacerbates neuroinflammation along with greater expression of CC chemokines to provide early CNS-infiltration of CD11b^+^Ly-6C^hi^ monocytes.

### Regulation of Ly-6C^hi^ monocyte differentiation in CD11c^hi^ DC-ablated mice

**Figure 3:**
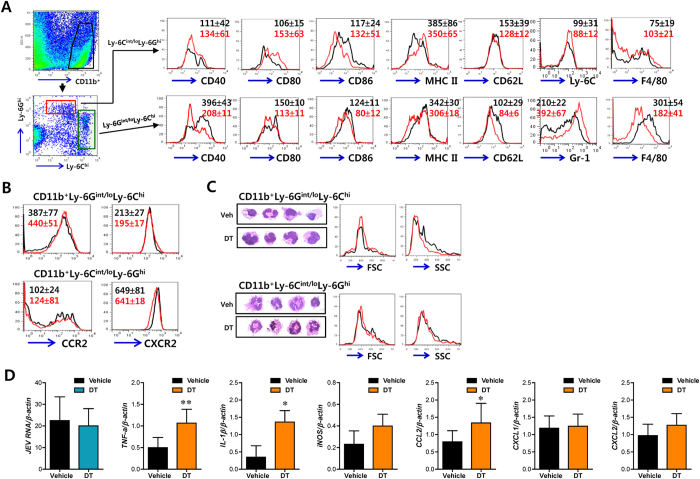
Regulation of Ly-6C^hi^ monocyte differentiation by CD11c^hi^ DCs. CD11c-DTR mice were injected i.p. with DT every other day from -1 to 7 days after JEV infection (1.5 × 10^7^ pfu/mouse). (**A**,**B**) The expression levels of differentiation markers and chemokine receptors in CNS-infiltrated CD11b^+^Ly-6C^hi^ monocytes and CD11b^+^Ly-6G^hi^ granulocytes. The expression levels of differentiation markers (**A**) and chemokine receptors (**B**) were determined by flow cytometric analysis at 3 dpi. The values in the histogram denote the average ± SD of MFI (*n* = 5–7). (**C**) Morphological examinations of CNS-infiltrated CD11b^+^Ly-6C^hi^ monocytes and CD11b^+^Ly-6G^hi^ granulocytes. CD11b^+^Ly-6C^hi^ monocytes and CD11b^+^Ly-6G^hi^ granulocytes were isolated at 3 dpi and stained with H&E. FSC and SSC histograms are representative of three individual experiments (*n* = 5–7). Black line, vehicle-treated; Red line, DT-treated group. (**D**) Levels of viral burden as well as cytokine and chemokine mRNAs in CD11b^+^Ly-6C^hi^ monocytes. The levels of viral burden and mRNAs for cytokines and chemokines were determined by real-time qRT-PCR using total RNA extracted from sorted CD11b^+^Ly-6C^hi^ monocytes at 3 dpi. Data denote the average levels ± SD of viral burden and cytokine mRNAs derived from at least three individual experiments (*n* = 3–4). *p < 0.05; **p < 0.01 compared with the levels of cells sorted from the CD11c-DTR mice group (vehicle) that received only JEV infection without DT injection (*n* = 5–7).

The debatable contribution of CD11b^+^Ly-6C^hi^ monocytes to immunopathology in the immune-privileged CNS has not been clearly defined^5^,^6^,^7^,^8^,^9^,^10^,^11^,^12^. The present finding implies that early infiltration of CD11b^+^Ly-6C^hi^ monocytes could be associated with severe neuroinflammatory reactions in immune-privileged CNS of CD11c^hi^ DC-ablated mice. Furthermore, since DC and monocytes share a common lineage during differentiation from HSCs^14^,^15^, it is presumable that CD11c^hi^ DC ablation affects the differentiation of CD11b^+^Ly-6C^hi^ monocytes during JE. To address this issue, we explored the impact of CD11c^hi^ DCs on the function and differentiation of CD11b^+^Ly-6C^hi^ monocytes during JE progression. Splenic CD11b^+^Ly-6G^hi^ neutrophils showed comparable levels in the presence or absence of CD11c^hi^ DCs, on evaluation of the expression levels of several molecules related to antigen presentation (CD40, CD62L, CD80, CD86, MHC I, MHC II), chemokine receptors (CCR2, CXCR2), and other differentiation markers (Ly-6C, Gr-1, F4/80). However, the expression levels of CD40, CD62L, CD80, CD86, MHC I, MHC II, and F4/80 in CD11b^+^Ly-6C^hi^ monocytes were decreased by CD11c^hi^ DC ablation (Fig. S5A). Notably, down-regulation of CD40, CD80, CD86, MHC I, MHC II, and F4/80 in CD11b^+^Ly-6C^high^ monocytes of DC-ablated mice was more apparent after JEV infection, compared to those of JEV-infected CD11c-DTR mice that were not injected with DT. In line with this result, the expression levels of CD40, CD62L, CD80, CD86, MHC I, MHC II, and F4/80 in CD11b^+^Ly-6C^hi^ monocytes infiltrated into immune-privileged CNS were markedly decreased in DC-ablated mice (Fig. 3A). On the other hand, the expression levels of chemokine receptors CXCR2 in Ly-6C^hi^ monocytes and Ly-6G^hi^ granulocytes were not significantly changed by CD11c^hi^ DC-depletion, whereas increased expression of CCR2 molecule was observed in CNS-infiltrated CD11b^+^Ly-6C^hi^ monocytes of CD11c^hi^ DC-depleted mice (MFI 440 ± 51 vs 387 ± 77) (Fig. 3B). The down-regulation of activation and differentiation markers in CD11b^+^Ly-6C^hi^ monocytes of CD11c^hi^ DC-ablated mice was also confirmed using recipient mice reconstituted by BM cells derived from CD11c-DTR mice, in order to assure the residual effect of DT (Fig. S6). Additionally, it was revealed that the expression levels of differentiation markers in CD11b^+^Ly-6C^hi^ monocytes and CD11b^+^Ly-6G^hi^ neutrophils of BM cells were not affected by the depletion of CD11c^hi^ DCs (Fig. S5B), which indicates that the differentiation of CD11b^+^Ly-6C^hi^ monocytes in the spleen and brain, but not BM, is affected by CD11c^hi^ DC ablation.

Because some CD11b^+^Ly-6C^hi^ monocytes could be differentiated into CD11c-expressing monocyte-derived DCs during inflammation^15^,^16^,^17^, thereby affecting the expression of costimulatory and MHC class II molecules in CD11b^+^Ly-6C^hi^ monocytes, we examined the frequency and number of CD11b^+^Ly-6C^hi^ monocytes expressing CD11c molecule in the fraction of CD11b^+^Ly-6C^hi^ monocytes. As expected, a small fraction of splenic CD11b^+^Ly-6C^hi^ monocyte expressing CD11c molecule was moderately reduced by DT injection (Fig. S7A). However, the frequency and number of CD11c-expressing CD11b^+^Ly-6C^hi^ monocytes infiltrated into the CNS were not significantly altered by DT injection, due to higher CNS-infiltration of CD11b^+^Ly-6C^hi^ monocytes in CD11c^hi^ DC-ablated mice (Fig. S7B). Therefore, it was thought that CD11c-expressing monocytes might affect the expression of MHC II and costimulatory molecules in CD11b^+^Ly-6C^hi^ monocytes, due to highly dominant population of CD11c-negative Ly-6C^hi^ monocytes. It is believed that CD11b^+^Ly-6C^hi^ monocytes develop in BM and arise from cMoP derived from MDP^13^,^14^. Therefore, to further examine the impact of CD11c^hi^ DCs on the differentiation of CD11b^+^Ly-6C^hi^ monocytes, we examined the frequency and number of monocyte progenitors, MDP and cMoP, in BM of CD11c-DTR mice during JE (Fig. S7C). CD11c^hi^ DC ablation provided slightly increased frequency, but not number, of MDP in BM before infection, while cMoP was accumulated in BM only during JE. Of note, CD11c^hi^ DC-ablated mice contained moderately decreased number of cMoP in BM compared to vehicle-treated mice, which implies that the differentiation process of MDP into cMoP could be affected by CD11c^hi^ DC ablation. To further characterize the regulation of CD11b^+^Ly-6C^hi^ monocyte differentiation by CD11c^hi^ DCs, we next examined their morphologies (Fig. 3C). Based on morphological examinations of monocytes described by Goasguen *et al.*^30^, the morphology of CD11b^+^Ly-6C^hi^ monocytes in DC-ablated mice come near to immature monocytes, compared to CD11b^+^Ly-6C^hi^ monocytes in vehicle-treated CD11c-DTR mice. CD11b^+^Ly-6C^hi^ monocytes in DC-ablated mice showed a monotonous shape for the cell membrane and a round/oval nuclear shape, whereas CD11b^+^Ly-6C^hi^ monocytes in vehicle-treated CD11c-DTR mice showed irregular shape of cell membrane and lobulated/indented nuclear shape which is the typical morphology of a mature monocyte and/or macrophage-like morphology. This immature morphology for the CD11b^+^Ly-6C^hi^ monocytes in DC-ablated mice was also supported by FSC and SSC results of flow cytometric analysis (Fig. 3C). Collectively, these results suggest that CD11c^hi^ DCs play a certain role in regulating the differentiation of CD11b^+^Ly-6C^hi^ monocytes during JE, thereby affecting the outcome of JE progression.

### CD11c^hi^ DCs maintain phenotypic homeostasis of CD11b^+^Ly-6C^hi^ monocytes

**Figure 4:**
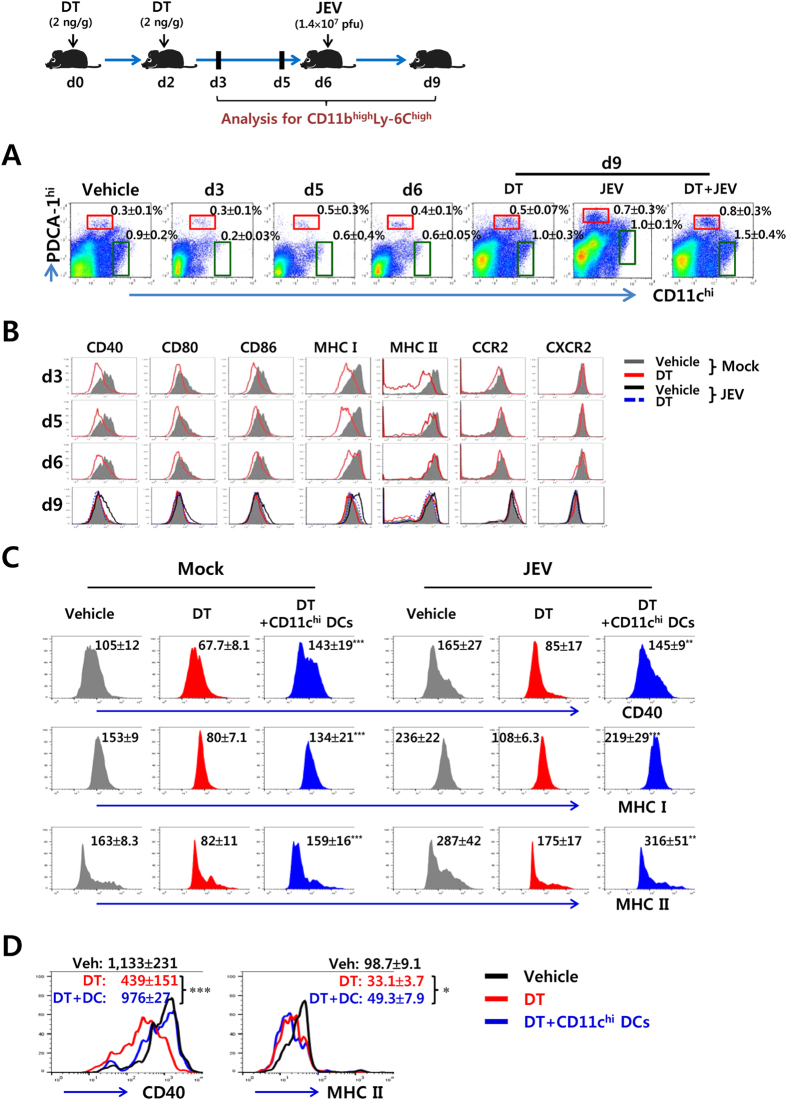
CD11c^hi^PDCA-1^int/lo^ DCs regulate phenotypic homeostasis of CD11b^+^Ly-6C^hi^ monocytes. CD11c-DTR mice were injected i.p. with DT (2 ng/g) 2 times at one-day interval, and the activation levels of splenic CD11b^+^Ly-6C^hi^ monocytes were analyzed 1 (d3), 3 (d5), and 4 (d6) days after last DT injection. Some mice were infected with JEV (1.5 × 10^7^ pfu/mouse) 4 days after last DT injection and the activation levels of monocytes were analyzed 3 days later (d9). (**A**) Changes of the proportion of CD11c^hi^PDCA-1^int/lo^ and CD11c^int^PDCA-1^hi^ DCs. The values of representative dot-plots denote the average percentage ± SD of the indicated cell population derived from at least four mice per group. (**B**) The activation levels of splenic CD11b^+^Ly-6C^hi^ monocytes. The histograms are representative of at least four individual experiments (*n* = 3–4). (**C**,**D**) Recovery of phenotypic levels of CD11b^+^Ly-6C^hi^ monocytes by CD11c^hi^PDCA-1^int/lo^ DCs. CD11c-DTR mice injected with DT every other day from -1 to 7 days after JEV infection were given the injection of CD11c^hi^PDCA-1^int/lo^ DCs (1.0 × 10^6^ cells/mouse) sorted from the spleen of BL/6 mice at the 2 dpi, and the phenotypic levels of CD11b^+^Ly-6C^hi^ monocytes in the spleen (**C**) and brain (**D**) of CD11c-DTR recipients were determined by flow cytometric analysis 2 days later. The values of representative histograms denote the average MFI ± SD of the indicated marker in CD11b^+^Ly-6C^hi^ monocytes derived from at least three individual experiments (*n* = 3–4). The pictures of mice were drawn by S.K. Eo. *p < 0.05; **p < 0.01; ***p < 0.001 compared with CD11b^+^Ly-6C^hi^ monocytes of DT-treated CD11c-DTR mice.

We next examined viral burden and cytokine expression of CD11b^+^Ly-6C^hi^ monocytes accumulated in the CNS of vehicle or DT-treated CD11c-DTR mice during JE progression (Fig. 3D). There was no significant difference in viral burdens of CD11b^+^Ly-6C^hi^ monocytes derived from CD11c-DTR mice that CD11c^hi^ DCs were depleted or not. However, infiltrated CD11b^+^Ly-6C^hi^ monocytes in the CNS of DC-ablated mice showed higher expression of some inflammatory cytokines than those of CD11c-DTR mice in which CD11c^hi^ DCs were not depleted. Notably, the expression levels of TNF-α and CCL2, which showed prominent increases in the CNS during JE progression, were significantly increased in CD11b^+^Ly-6C^hi^ monocytes infiltrated into the immune-privileged CNS of DC-ablated mice, compared to those of vehicle-treated CD11c-DTR mice (Fig. 3D). Also, in general, CD11c^hi^ DC number was gradually restored within 3–4 days after stopping injection of DT (Fig. 4A). Thus, we were interested in whether the restoration of CD11c^hi^ DCs after stopping DT injection could induce the phenotypic recovery of CD11b^+^Ly-6C^hi^ monocytes (Fig. 4B). After stopping DT injection, the expression levels of differentiation markers in CD11b^+^Ly-6C^hi^ monocytes were gradually recovered until reaching the levels found in CD11b^+^Ly-6C^hi^ monocytes in vehicle-treated CD11c-DTR mice, depending on restoration of CD11c^hi^ DCs. Of note, the phenotypic recovery of CD11b^+^Ly-6C^hi^ monocytes was faster if CD11c-DTR mice were infected with JEV. Furthermore, to confirm the phenotypic recovery of CD11b^+^Ly-6C^hi^ monocytes by CD11c^hi^ DC restoration, we tested if down-regulated phenotype of CD11b^+^Ly-6C^hi^ monocytes in CD11c^hi^ DC-ablated mice could be recovered by injection of sorted CD11c^hi^PDCA-1^int/lo^ DCs during JE (Fig. 4C,D). As expected, our results revealed that the injection of sorted CD11c^hi^ DCs provided the recovered phenotypes of CD11b^+^Ly-6C^hi^ monocytes in the spleen and CNS of DT-treated CD11c-DTR mice. Ultimately, these results suggest that CD11c^hi^ DCs play a role in maintaining phenotypic homeostasis of CD11b^+^Ly-6C^hi^ monocytes, and thereby affect neuroinflammation after their infiltration in the immune-privileged CNS.

### Flt3-L and GM-CSF are involved in Ly-6C^hi^ monocyte differentiation in DC-ablated mice

**Figure 5:**
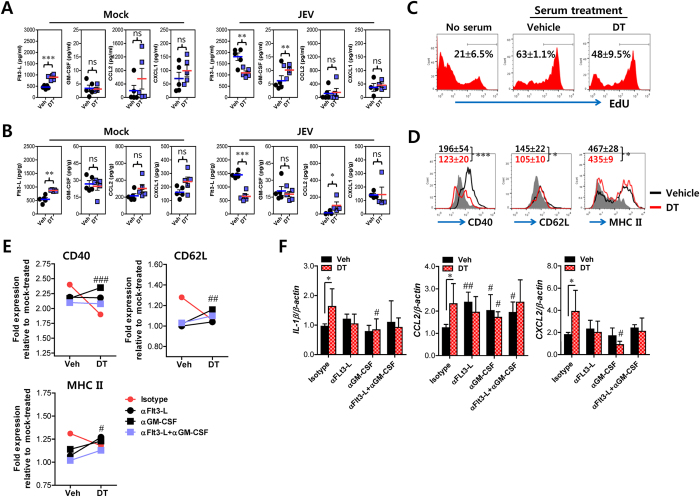
Flt3-L and/or GM-CSF play a role in regulating the differentiation of CD11b^+^Ly-6C^hi^ monocytes in CD11c^hi^ DC-depleted mice following JEV infection. CD11c-DTR mice were injected i.p. with DT -1 and 1 day after JEV infection. Sera were collected from vehicle- or DT-treated mice at 3 dpi. (**A**,**B**) Levels of hematopoietic factors and chemokines in sera and spleen. The levels of hematopoietic factors and chemokines in sera (**A**) and spleen (**B**) collected at 3 dpi were determined by ELISA and CBA methods. Each symbol represents levels of individual mouse; horizontal line indicates median of each group (*n* = 5). (**C**) Proliferation of CD11b^+^Ly-6C^hi^ monocytes by sera. Splenocytes prepared from DC-depleted mice were incubated with sera derived from JEV-infected CD11c-DTR 3 dpi. Proliferation of CD11b^+^Ly-6C^hi^ monocytes was assessed by EdU incorporation after 48-h incubation. The values in representative histogram denote the average ± SD of % EdU-positive cells after gated on CD11b^+^Ly-6C^hi^ monocytes in quadruplicate wells. (**D**) Phenotypic changes in CD11b^+^Ly-6C^hi^ monocytes by sera. After 48-h incubation of DC-depleted splenocytes with sera, the expression of phenotypic markers was determined by flow cytometric analysis. The values in representative histogram denote the average ± SD of MFI of CD11b^+^Ly-6C^hi^ monocytes in quadruplicate wells. The gray line represents the expression levels of phenotypic markers in serum-untreated CD11b^+^Ly-6C^hi^ monocytes. (**E**) Recovery of under-differentiated CD11b^+^Ly-6C^hi^ monocytes by blocking Flt3-L and GM-CSF. Splenocytes prepared from DC-depleted mice were incubated with sera in the presence of anti-Flt3-L and/or anti-GM-CSF antibodies for 48 h. Data represent fold changes in the expression level of each phenotype marker relative to those of serum-untreated CD11b^+^Ly-6C^hi^ monocytes. (**F**) Cytokine expression by CD11b^+^Ly-6C^hi^ monocytes in the presence of anti-Flt3-L and GM-CSF antibodies. After 48-h incubation of DC-depleted splenocytes with sera in the presence of anti-Flt3-L and -GM-CSF antibodies, the expression levels of mRNAs of cytokines and chemokines were determined by real-time qRT-PCR using total RNA extracted from sorted CD11b^+^Ly-6C^hi^ monocytes. Data represent the average ± SD of mRNA levels derived from monocytes in quadruplicate wells. *p < 0.05; **p < 0.01; ***p < 0.001 between vehicle and DT-treated groups. ^#^*p* < 0.05; ^##^*p* < 0.01 compared to the corresponding isotype-treated group. ns, Not significant.

A previous study showed that mice with conditional DC depletion develop myeloproliferative disorder (MDP), as indicated by a massive increase in CD11b^+^ cell number with elevated serum levels of Flt3-L^31^. In the current study, we also found elevated Flt3-L levels in sera and spleen of CD11c^hi^ DC-depleted mice, while changes in GM-CSF, CCL2, and CXCL1 were not observed (Fig. 5A,B). However, such levels of Flt3-L in serum and spleen were dramatically enhanced in vehicle-treated CD11c-DTR mice following JEV infection, rather than in CD11c^hi^ DC-depleted mice. Interestingly, however, the GM-CSF level in serum, but not the spleen, was significantly enhanced in CD11c^hi^ DC-depleted mice following JEV infection. Because inadequate differentiation of CD11b^+^Ly-6C^hi^ monocytes in DC-ablated mice was more apparent after JEV infection along with alteration of Flt3-L and GM-CSF production and the proliferation and phenotypes of CD11b^+^Ly-6C^hi^ monocytes were not significantly altered by incubation with sera derived from uninfected CD11c-DTR mice that received DT injection (Fig. S8), we were focused on the roles of serum Flt3-L and GM-CSF in modulating the differentiation of CD11b^+^Ly-6C^hi^ monocytes using sera obtained from JEV-infected CD11c-DTR mice that were previously treated with vehicle or DT. Our results revealed that moderately elevated proliferation of CD11b^+^Ly-6C^hi^ monocytes was achieved by incubating the splenocytes of DC-ablated mice with sera of vehicle-treated CD11c-DTR mice (Fig. 5C), while decreased expression of surface markers CD40, CD62L, and MHC class II in CD11b^+^Ly-6C^hi^ monocytes was observed in sera obtained from CD11c^hi^ DC-depleted mice (Fig. 5D). In addition, in order to examine whether Flt3-L and GM-CSF were involved in such down-regulation of CD40, CD62L, and MHC class II, the splenocytes of CD11c^hi^ DC-depleted mice were incubated with sera in the presence of neutralizing Abs against Flt3-L and GM-CSF. The neutralization of Flt3-L and/or GM-CSF induced significantly recovered expression levels of CD40, CD62L, and MHC class II in CD11b^+^Ly-6C^hi^ monocytes incubated with sera derived from CD11c^hi^ DC-depleted mice (Fig. 5E). This finding indicates that Flt3-L and GM-CSF could modulate the down-regulation of differentiation markers in CD11b^+^Ly-6C^hi^ monocytes derived from CD11c^hi^ DC-depleted mice. Also, we examined the expression levels of cytokines and chemokines in CD11b^+^Ly-6C^hi^ monocytes incubated with sera derived from JEV-infected CD11c-DTR mice that received vehicle or DT. CD11b^+^Ly-6C^hi^ monocytes incubated with sera of CD11c^hi^ DC-depleted mice showed higher expression levels of IL-1β, CCL2, and CXCL2, compared to monocytes incubated with sera of vehicle-treated mice (Fig. 5F). Such increased expression levels of cytokines and chemokines in CD11b^+^Ly-6C^hi^ monocytes incubated with sera of DC-ablated mice were significantly reduced by blocking GM-CSF, whereas lower expression levels of cytokines and chemokines in CD11b^+^Ly-6C^hi^ monocytes incubated with sera of vehicle-treated mice were reversed or moderately enhanced with treatment of anti-Flt3-L neutralizing antibody. Of note, CCL2 expression in CD11b^+^Ly-6C^hi^ monocytes incubated with sera of vehicle-treated CD11c-DTR mice was significantly enhanced by blocking Flt3-L and/or GM-CSF. Collectively, these results imply that down-regulation of differentiation markers and enhanced expression of inflammatory cytokine and chemokine in CD11b^+^Ly-6C^hi^ monocytes derived from DC-ablated mice could be mediated by Flt3-L and GM-CSF with their differential trends following JEV infection.

### Ly-6C^hi^ monocytes differentiated in CD11c^hi^ DC-ablated environment augment neuroinflammation in immune-privileged CNS

**Figure 6:**
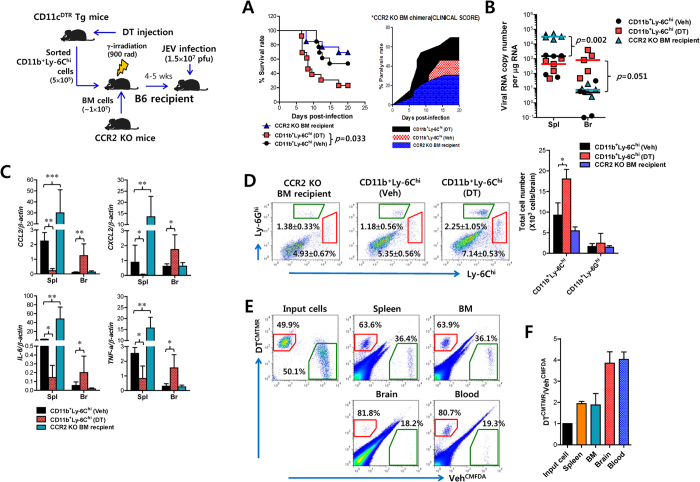
Ly-6C^hi^ monocytes generated in CD11c^hi^ DC–ablated environment exacerbate neuroinflammation. BM cells isolated from CCR2 KO mice were injected into γ-irradiated B6 recipients (*n* = 13), after which the recipients received adoptive transfer of CD11b^+^Ly-6C^hi^ monocytes (1.5 × 10^6^ cells/mouse) sorted from DT- or vehicle-injected CD11c-DTR mice, followed by JEV infection. (**A**) Survival and paralysis rates. Proportion of mice that survived and showed paralysis was monitored until 20 dpi. (**B**,**C**) Viral burden and expression of cytokine and chemokine mRNAs. Viral burden and expression of cytokines and chemokines in the spleen (Spl) and brain (Br) of CCR2 KO BM recipients that received CD11b^+^Ly-6C^hi^ monocytes were assessed by real-time qRT-PCR at 3 dpi. Each symbol represents viral burden of individual mouse; horizontal line indicates median of each group. Bar charts denote the average level ± SD of cytokine and chemokine mRNAs per group (*n* = 5). (**D**) Infiltrated CD11b^+^Ly-6C^hi^ monocytes in CCR2 KO BM recipients. The frequencies and absolute numbers of CD11b^+^Ly-6C^hi^ monocytes and CD11b^+^Ly-6G^hi^ granulocytes were analyzed by flow cytometric analysis 3 days after adoptive transfer of sorted CD11b^+^Ly-6C^hi^ monocytes and JEV infection. Dot-plots show representative after gated on CD11b^+^ cells. Bar graph shows the average ± SD of indicated cell number obtained from three individual experiments (*n* = 4–5). (**E**) Enhanced accumulation in the CNS and lymphoid tissues of CD11b^+^Ly-6C^hi^ monocytes sorted from DT-treated CD11c-DTR. Sorted and differentially labeled (Veh^CMFDA^ and DT^CMTMR^) CD11b^+^Ly-6C^hi^ monocytes from vehicle- or DT-treated CD11c-DTR were injected into JEV-infected CCR2 KO mice with equal ratio at 4 dpi. The accumulation of CD11b^+^Ly-6C^hi^ monocytes in the CNS and lymphoid tissues was determined by flow cytometric analysis 20 h after cell injection. Dot-plot is representative of four individual experiments and values of the dot-plot are average percentages of Veh^CMFDA^ and DT^CMTMR^. (**F**) Distribution ratio of labeled CD11b^+^Ly-6C^hi^ monocytes from DT- and vehicle-treated CD11c-DTR mice. Total number of accumulated CD11b^+^Ly-6C^hi^ in each tissue (*n* = 4) was determined at 4 dpi, and then the distribution ratio of labeled CD11b^+^Ly-6C^hi^ from vehicle- and DT-treated CD11c-DTR mice was assessed. The pictures of mice were drawn by S.K. Eo. *p < 0.05; **p < 0.01; ***p < 0.001 compared with the levels of the indicated group.

It is still unclear whether infiltration of CD11b^+^Ly-6C^hi^ monocytes in immune-privileged CNS plays a deleterious or beneficial role in neuroinflammation caused by neurotropic viruses such as WNV^5^,^6^,^7^,^8^,^9^,^10^,^11^,^12^. Although our results propose early pronounced CNS infiltration of inadequately differentiated CD11b^+^Ly-6C^hi^ monocytes in the absence of CD11c^hi^ DCs, resulting in the induction of severe neuroinflammation and a deleterious role in encephalitis, we did not provide direct evidence as to whether infiltration of inadequately differentiated CD11b^+^Ly-6C^hi^ monocytes into the immune-privileged CNS played a deleterious or beneficial role in JE progression. To address this issue, we used recipient mice reconstituted by BM cells derived from CCR2 KO mice that are monocytopenic, in which tissue recruitment of monocytes is impaired in infectious disease models^9^. CCR2 KO BM recipients were infected with JEV after adoptive transfer of sorted CD11b^+^Ly-6C^hi^ monocytes from DT- or vehicle-injected CD11c-DTR mice. Interestingly, CCR2 KO BM recipients that received CD11b^+^Ly-6C^hi^ monocytes derived from DC-ablated mice were more susceptible to JE, as compared to CCR2 KO BM recipients given CD11b^+^Ly-6C^hi^ monocytes sorted from vehicle-treated CD11c-DTR mice (*p* = 0.033) (Fig. 6A). There was also a moderate increase in susceptibility to JE in CCR2 KO BM recipients given CD11b^+^Ly-6C^hi^ monocytes from vehicle-treated mice, compared to JEV-infected CCR2 KO BM recipients given no monocytes. Furthermore, CCR2 KO BM recipients that received CD11b^+^Ly-6C^hi^ monocytes sorted from CD11c^hi^ DC-depleted mice showed faster development of neurological disorders after JEV infection than did recipients of CD11b^+^Ly-6C^hi^ monocytes from vehicle-treated mice. We also used recipient mice reconstituted by BM cells derived from CD11c-DTR·CCR2 KO mice to exclude the recovery potential of inadequately differentiated CD11b^+^Ly-6C^hi^ monocytes by endogenous CD11c^hi^ DCs (Fig. S9). The recipients of BM cells derived from CD11c-DTR·CCR2 KO mice were infected with JEV following the adoptive transfer of CD11b^+^Ly-6C^hi^ monocytes sorted from CD11c-DTR mice that received DT or vehicle. CD11c-DTR·CCR2 KO BM recipients were injected with DT to inhibit the recovery potential of adoptively transferred CD11b^+^Ly-6C^hi^ monocytes by endogenous CD11c^hi^ DCs. Similar to the experiment using CCR2 KO BM recipients, CD11c-DTR·CCR2 KO BM recipients that received CD11b^+^Ly-6C^hi^ monocytes sorted from CD11c^hi^ DC-ablated mice showed higher susceptibility to JE than did the recipients of CD11b^+^Ly-6C^hi^ monocytes sorted from vehicle-treated CD11c-DTR mice, as evaluated by mortality (*p* = 0.0349) (Fig. S9A), rapid development of neurological disorder (Fig. S9B), and body weight change during JE progression (Fig. S9C). Therefore, these results provide direct evident that inadequately differentiated CD11b^+^Ly-6C^hi^ monocytes generated in CD11c^hi^ DC-ablated mice are more deleterious to neuroinflammation than those of vehicle-treated CD11c-DTR mice, thereby providing exacerbated JE.

To better understand the augmented JE of CCR2 KO BM recipients given CD11b^+^Ly-6C^hi^ monocytes from DC-ablated mice, we measured viral burden in the spleen and brain of JEV-infected CCR2 KO BM recipients (Fig. 6B). CCR2 KO BM recipients that received just JEV infection without the supply of CD11b^+^Ly-6C^hi^ monocytes showed the highest viral burden in the spleen as the primary target tissue of virus administered *via* i.p. route, compared to CCR2 KO BM recipients that received adoptive transfer of CD11b^+^Ly-6C^hi^ monocytes derived from CD11c-DTR mice given DT or vehicle. However, interestingly, CCR2 KO BM recipients given sorted CD11b^+^Ly-6C^hi^ monocytes from CD11c^hi^ DC-depleted mice had significantly increased levels of viral burden in the immune-privileged CNS tissues, compared to CCR2 KO BM recipients that received CD11b^+^Ly-6C^hi^ monocytes sorted from vehicle-treated CD11c-DTR mice or just JEV-infected CCR2 KO BM recipients. Also, the expression levels of pro-inflammatory cytokines (TNF-α and IL-6) and chemokines (CCL2 and CXCL2) were closely associated with the levels of viral burden in the spleen and the CNS (Fig. 6C). Collectively, these results indicate that CD11b^+^Ly-6C^hi^ monocytes derived from DC-ablated mice exacerbate neuroinflammation in immune-privileged CNS during JE progression by supplying enhanced viral burden and expression of pro-inflammatory cytokines.

### Ly-6C^hi^ monocytes generated in CD11c^hi^ DC-ablated environment are accumulated faster in inflamed CNS, depending on CCR2

Next, to further define the exacerbation of neuroinflammation by CD11b^+^Ly-6C^hi^ monocytes derived from DC-ablated mice, we examined the infiltration of CD11b^+^Ly-6C^hi^ monocytes in the CNS of CCR2 KO BM recipients. As expected, infiltrated CD11b^+^Ly-6C^hi^ monocytes in the CNS were observed with higher frequency and absolute number in the immune-privileged CNS of CCR2 KO BM recipients that received CD11b^+^Ly-6C^hi^ monocytes sorted from DC-ablated mice, compared to other recipients (Fig. 6D). Therefore, this more rapid CNS accumulation in CCR2 KO BM recipients of CD11b^+^Ly-6C^hi^ monocytes sorted from DC-depleted mice was closely associated with enhanced viral burden and cytokine expression.

To trace trafficking of adoptively transferred CD11b^+^Ly-6C^hi^ monocytes in the CNS, we analyzed monocyte trafficking from blood to CNS, spleen, and BM directly using a competitive study where equal numbers of CD11b^+^Ly-6C^hi^ monocytes derived from DT- or vehicle-treated CD11c-DTR mice were differentially labeled (Veh^CMFDA^ vs DT^CMTMR^), mixed 1:1, and injected into JEV-infected CCR2 KO recipient mice. Generally, a skewed distribution favoring CD11b^+^Ly-6C^hi^ monocytes (DT^CMTMR^) derived from CD11c^hi^ DC-depleted mice was observed in lymphoid (spleen and BM) and inflammatory tissue (brain) (Fig. 6E). Notably, donor DT^CMTMR^ cells displayed a more dramatically skewed distribution in blood and inflamed CNS, compared to lymphoid tissues such as spleen and BM. In agreement with this finding, the ratios of absolute numbers of DT^CMTMR^ to Veh^CMFDA^ were greater in blood and CNS (~4:1) than those of spleen and BM (~2:1) (Fig. 6F). Taken together, these results indicate that CD11b^+^Ly-6C^hi^ monocytes differentiated in the absence of CD11c^hi^ DCs migrate to the immune-privileged CNS with a skewed distribution to induce severe neuroinflammatory reaction in a CCR2-dependent manner.

### Decreased JEV-specific humoral and T cell responses in CD11c^hi^ DC-ablated mice

**Figure 7:**
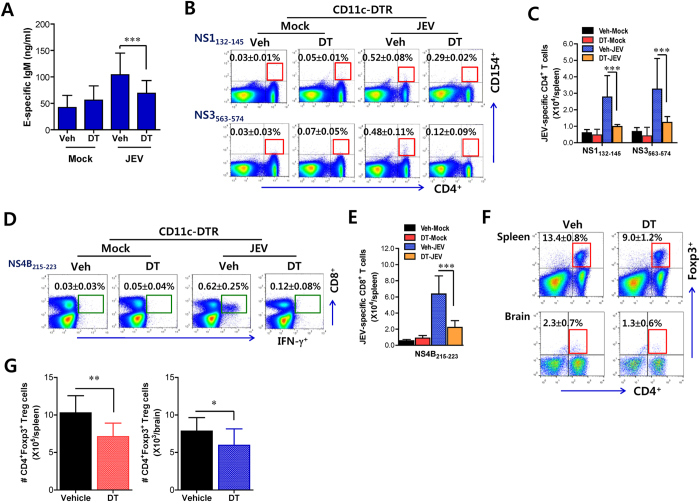
Decreased JEV-specific humoral and T cell responses in CD11c^hi^ DC-ablated mice. CD11c-DTR mice were injected i.p. with DT every other day from -1 to 7 days after JEV infection (1.5 × 10^7^ pfu/mouse). (**A**) JEV E-specific IgM response. Sera were collected from survived mice at 7 dpi and used for ELISA to detect IgM levels specific for JEV E protein. Data shows the average ± SD of JEV E-specific IgM levels derived from survived mice (*n* = 6–13). (**B**,**C**) CD4^+^ T cell response specific for JEV antigen. The splenocytes prepared from survived mice (*n* = 4–5) were stimulated with JEV epitope peptides of CD4^+^ T cells (NS1_132−145_ and NS3_563−574_) for 12 h. The frequency (**B**) and absolute number (**C**) of CD4^+^ T cells specific for JEV epitope peptides were determined by intracellular CD154 staining, combined with surface CD4 staining. (**D**,**E**) CD8^+^ T cell response specific for JEV antigen. The frequency (**D**) and absolute number (**E**) of CD8^+^ T cells specific for JEV epitope peptide (NS4B_215−223_) were determined by intracellular IFN-γ staining after a 8-h stimulation with peptide. (**F**,**G**) Impact of CD11c^hi^ DC ablation on CD4^+^Foxp3^+^ Treg cells. The frequency (**F**) and absolute number (**G**) of CD4^+^Foxp3^+^ Treg cells in the spleen and brain were determined by flow cytometric analysis using survived mice 5 dpi. The values in representative dot-plots and bar charts represent the average ± SD of CD4^+^/CD8^+^ T cell responses and CD4^+^Foxp3^+^ Treg cells per group (*n* = 4–5). *p < 0.05; **p < 0.01; ***p < 0.001 compared with the levels of the indicated group.

Although CD11b^+^Ly-6C^hi^ monocytes generated in the absence of CD11c^hi^ DCs exacerbated JE, our data might discount the role of JEV-specific adaptive immune responses in JE progression. Because the role of CD11c^hi^ DCs in initiating Ag-specific CD4^+^ and CD8^+^ T-cell responses have been well defined, it is expected that CD11c^hi^ DC-ablated mice could develop decreased humoral and T cell responses specific for JEV Ag. Although CD11c-DTR mice infected with JEV usually exhibited neurological disorder at 3–4 dpi before fully functional adaptive immune responses are induced, we examined JEV-specific humoral and T-cell responses using survived mice at 7 dpi. As expected, decreased levels of JEV E protein-specific IgM were observed in sera of CD11c^hi^PDCA-1^int/lo^ DC-depleted mice (Fig. 7A). Similarly, DC-ablated mice showed decreased percentages and absolute number of JEV-specific CD4^+^ T cells, as evaluated by intracellular CD154 staining upon stimulation with two epitope peptides of CD4^+^ T cells (Fig. 7B,C). Furthermore, diminished percentage and absolute number of IFN-γ-producing CD8^+^ T cells in response to stimulation with JEV CD8^+^ T cell epitope peptide were observed in CD11c^hi^ DC-depleted mice (Fig. 7D,E). Since CD4^+^Foxp3^+^ Treg cells may contribute to control neuroinflammation caused by neurotropic virus^32^, we also addressed the frequency and number of CD4^+^Foxp3^+^ Treg cells in the spleen and brain of survived mice 5 dpi. CD11c^hi^ DC-depleted mice exhibited decreased frequency and absolute number of CD4^+^Foxp3^+^ Treg cells in the spleen and brain (Fig. 7F,G), which indicates that decreased CD4^+^Foxp3^+^ Treg cells could contribute to exacerbation of JE in CD11c^hi^ DC-depleted mice. Therefore, these results suggest that decreased JEV-specific humoral and T-cell responses and CD4^+^Foxp3^+^ Treg cells provide failure to control severe neuroinflammation in DC-ablated mice, thereby more exacerbating JE at later phase.

### CD11c^hi^PDCA-1^int/lo^ DCs do not affect the spread of JEV in the brain after intracranial inoculation

**Figure 8:**
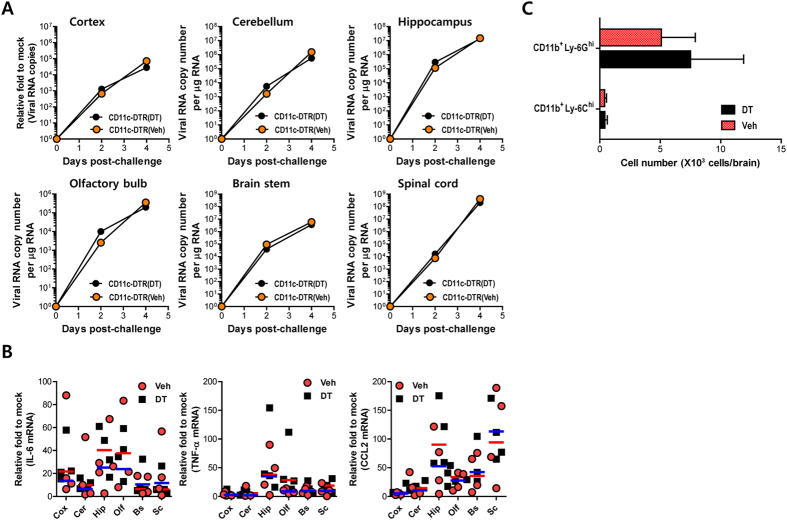
A deficiency of CD11c^hi^ DCs does not facilitate the spread of JEV in the brain after intracranial inoculation. CD11c-DTR mice were injected i.p. with DT every other day from -1 to 7 days after intracranial JEV infection (1.5 × 10^4^ pfu/mouse). (**A**) Viral burden. The CNS tissues were separated into cerebral cortex (Cox), cerebellum (Cer), hippocampus (Hip), olfactory bulb (Olf), brain stem (Bs), and spinal cord (Sc) at the indicated dpi. Viral burden was determined by real-time qRT-PCR using JEV-specific primers. Data was expressed as viral RNA copy number per microgram of total RNA (*n* = 4–5). (**B**) Levels of pro-inflammatory cytokines and chemokines in the sub-CNS tissues. The levels of each cytokine were determined by real-time qRT-PCR 2 dpi. Each symbol represents a level of an individual mouse; horizontal line indicates the median of each group. (**C**) Accumulation of CD11b^+^Ly-6C^hi^ monocytes and CD11b^+^Ly-6G^hi^ granulocytes in the brain. After vigorous heart perfusion on the 2nd dpi, total number of CD11b^+^Ly-6C^hi^ monocytes and CD11b^+^Ly-6G^hi^ granulocytes infiltrated into the brain were analyzed by flow cytometric analysis. Data represent the average ± SD derived from six mice per group.

Although virus burden in the CNS of CD11c^hi^PDCA-1^int/lo^ DC-depleted mice was elevated after JEV infection, it was unclear whether the depletion of peripheral CD11c^hi^PDCA-1^int/lo^ DCs directly affected the viral spread in the CNS. Moreover, in consistent with previous reports^20^,^21^, DCs in the CNS were not affected by DT injection (data not shown). Thus, to determine whether CD11c^hi^PDCA-1^int/lo^ DC-depletion facilitated the direct spreading of virus within the CNS, we directly evaluated JEV replication in several different sub-tissues of the CNS (cortex, cerebellum, hippocampus, olfactory bulb, brain stem, and spinal cord) 2 and 4 days after direct intracranial infection. The viral burden in several different sub-tissues of brain was not changed by the depletion of CD11c^hi^PDCA-1^int/lo^ DCs at 2 and 4 days after intracranial infection (Fig. 8A), which indicates that the depletion of peripheral CD11c^hi^PDCA-1^int/lo^ DCs does not affect direct CNS dissemination of the virus. This implies that the amount of delivered virus from the periphery may contribute to the enhancement of viral burden in the CNS of DC-ablated mice. Also, CD11c^hi^PDCA-1^int/lo^ DC-depleted mice showed no significant change in the expression of pro-inflammatory cytokine mRNAs in several different sub-tissues of brain intracranially infected with JEV, compared to vehicle-treated mice (Fig. 8B). Furthermore, the infiltration levels of CD11b^+^Ly-6C^hi^ monocytes and CD11b^+^Ly-6G^hi^ neutrophils after intracranial infection of JEV were comparable in both vehicle- and DT-treated CD11c-DTR mice (Fig. 8C). Collectively, these results indicate that the peripheral depletion of CD11c^hi^PDCA-1^int/lo^ DCs exerts no influence on neuronal dissemination of virus inoculated within the CNS.

## Discussion

Our data demonstrate that CD11c^hi^PDCA-1^int/lo^ DCs, but not CD11c^int^PDCA-1^hi^ pDCs, are essential to control lethal neuroinflammation through regulating the infiltration of CD11b^+^Ly-6C^hi^ monocytes and the expression of CC hemokines in the CNS. More interestingly, CD11b^+^Ly-6C^hi^ monocytes were found to have a deleterious rather than protective role during JE progression, and CD11b^+^Ly-6C^hi^ monocytes generated in CD11c^hi^ DC-ablated environment showed altered differentiation levels and exacerbated the progression of neuroinflammation *via* their faster and increased recruitment and subsequently enhanced supply of virus into inflamed CNS in a CCR2-dependent manner. Furthermore, Flt3-L and GM-CSF played a role in controlling the differentiation of CD11b^+^Ly-6C^hi^ inflammatory monocytes in the absence of CD11c^hi^ DCs. Ultimately, our results are the first to show that CD11c^hi^ DCs maintain the phenotypic and functional homeostasis of CD11b^+^Ly-6C^hi^ monocytes to preserve immune-privileged CNS in immunopathology caused by neurotropic viruses.

CD11b^+^Ly-6C^hi^ monocytes are believed to play an important role during CNS inflammation as the circulating precursors of brain macrophages, DCs, and arguably microglia in various disease models, such as experimental autoimmune encephalomyelitis (EAE), viral encephalitis, and stroke^4^,^5^,^6^,^7^,^8^,^9^,^10^,^11^,^12^. However, their role in CNS inflammation caused by a neurotropic virus such as WNV has not been clearly delineated due to conflicting results^9^,^10^,^11^,^12^. One clear finding in the present study was that CD11b^+^Ly-6C^hi^ monocytes have a deleterious role and exacerbate CNS inflammation, as proven by the enhanced severity of CNS inflammation when sorted CD11b^+^Ly-6C^hi^ monocytes were injected into CCR2 KO BM recipients. Notably, considering that inadequately differentiated CD11b^+^Ly-6C^hi^ monocytes developed in CD11c^hi^ DC-ablated environment caused more significantly augmented severity of neuroinflammation, the differentiation levels of infiltrated CD11b^+^Ly-6C^hi^ monocytes in the CNS reflect to be one of important factors for regulating the progression of lethal neuroinflammation. This finding is strengthened by the recent result that peripheral DCs play an important role in functionally priming Ly-6C^hi^ monocytes to promote its tissue-specific function during inflammation^33^. However, it is still unclear how CD11b^+^Ly-6C^hi^ monocytes infiltrated in immune-privileged CNS of CD11c^hi^ DC-depleted mice exacerbated neuroinflammation. One evidence to explain this question was that CD11b^+^Ly-6C^hi^ monocytes developed in CD11c^hi^ DC-depleted mice could provide exacerbated neuroinflammation through higher expression levels of TNF-α and CCL2 than those of vehicle-treated mice, since TNF-α and CCL2 have been known to induce neuronal apoptosis, increase BBB permeability, and amplify infiltration of CD11b^+^Ly-6C^hi^ monocytes^34^,^35^. Also, DCs derived from blood CD11b^+^Ly-6C^hi^ monocytes showed distinct characteristics allowing them to induce neuroinflammation in the CNS, compared to DCs that resided in the CNS^36^. These additional pieces of evidence support our results. However, the higher expression of TNF-α and CCL2 in inadequately differentiated Ly-6C^hi^ monocytes during JE provides a bit opposite result that remains to be defined in future study. In addition, there was another incongruous finding regarding JEV RNA burden in CD11b^+^Ly-6C^hi^ monocytes derived from CD11c^hi^ DC-depleted mice. Our results revealed that infiltrated CD11b^+^Ly-6C^hi^ monocytes in the brains of vehicle- and DT-treated mice were comparable in viral RNA burden, which indicates that CD11b^+^Ly-6C^hi^ monocytes in both environments have a similar ability to deliver virus into the CNS. We also found that CD11b^+^Ly-6C^hi^ monocytes sorted from vehicle- and DT-treated mice showed no difference in permissiveness to *in vitro* viral infection (data not shown). One explanation to this incongruous finding is that increased number of CD11b^+^Ly-6C^hi^ monocytes in DC-ablated mice might be able to supply virus much more readily than those of CD11c-DTR mice in which CD11c^hi^ DCs were not depleted.

Intriguing finding in this study was that CD11c^hi^ DCs regulate the differentiation of CD11b^+^Ly-6C^hi^ monocytes. This regulation of Ly-6C^hi^ monocytes by DCs may be explained by mutually nonexclusive scenarios of increased Flt3-L and GM-CSF, because altered phenotype of CD11b^+^Ly-6C^hi^ monocytes is closely associated with elevated levels of Flt3-L and GM-CSF in the presence and absence, respectively, of CD11c^hi^ DCs following viral infection. First, since GM-CSF has been known to be an important factor in the control of CD11c^hi^ cDC development and maintenance at the periphery^37^, the ablation of CD11c^hi^ cDCs serving as a major “ligand sink” could provide elevated levels of GM-CSF in JEV-infected mice, due to decreased consumption of factors. Thus, elevated GM-CSF in DC-ablated mice is likely to provide immature monocytes during neuroinflammation progression. Alternatively, CD11c^hi^ DCs may also provide secreted or membrane-bound signals that regulate Flt3-L production by stromal cells or lymphocytes^38^. Considering that Flt3-L plays an important role in the development of CD11c^int^PDCA-1^hi^ pDCs as well as monocytes^39^, the elevated level of Flt3-L in vehicle-treated CD11c-DTR mice following JEV infection may stimulate the development of CD11c^int^PDCA-1^hi^ pDCs, as well as maturation of CD11b^+^Ly-6C^hi^ monocytes. Indeed, our results provided several clues to these possibilities; the increased number of CD11c^int^PDCA-1^hi^ pDCs after JEV infection along with elevated level of Flt3-L as well as the differential regulation of differentiation markers and CCL2 chemokine expression in CD11b^+^Ly-6C^hi^ monocytes by neutralization of Flt3-L and GM-CSF. In the absence of such a CD11c^hi^ DC-mediated feedback loop, altered levels of Flt3-L and GM-CSF would ultimately result in a changed phenotype and function of peripheral CD11b^+^Ly-6C^hi^ monocytes during JE progression. Furthermore, our data is supported by the report that GM-CSF can block Flt3-L-driven development of pDC by activating STAT5 that binds to IRF8 promoter^40^,^41^. Therefore, inadequate maturation of CD11b^+^Ly-6C^hi^ monocytes in selective ablation of CD11c^hi^ DCs is unlikely to result from a direct interplay between DCs and monocytes, but, instead, be related to imbalanced consumption of hematopoietic factors in DC-ablated model. Also, CD11c^hi^ DC ablation is likely to induce dysregulation of CD11b^+^Ly-6C^hi^ monocyte differentiation under steady state, because moderate alteration of CD11b^+^Ly-6C^hi^ monocyte differentiation has been already achieved in the spleen of DC-ablated mice before viral infection. It is thought that this inadequately differentiated CD11b^+^Ly-6C^hi^ monocytes in DC-ablated mice are rapidly deployed into the inflamed CNS following JEV infection^42^. Further elucidation of the molecular mechanism linking CD11c^hi^ DC loss to altered differentiation and function of CD11b^+^Ly-6C^hi^ monocytes may provide critical clues to understanding the differentiation lineage of DCs and monocytes.

Monocytes are derived from HSCs in BM via CD34^+^ common myeloid precursors (CMP), CD16/32^+^ granulocyte/macrophage precursors (GMPs), CD115^+^CD135^+^Ly-6C^−^ MDP, and then CD115^+^CD135^+^Ly-6C^+^ cMoP^13^,^14^. The spleen has also been identified as an important reservoir of monocytes that are rapidly deployed to sites of inflammation, including the ischemic heart and brain^42^. Whether the spleen is a significant source of CD11b^+^Ly-6C^hi^ monocytes during CNS inflammation caused by neurotropic viruses such as JEV and WNV is yet to be determined, but both spleen and BM are considered to be critical for supplying CD11b^+^Ly-6C^hi^ monocytes to the inflamed CNS, particularly in cases of acute and severe infection, in which large numbers of these cells are rapidly deployed and recruited to the brain^42^. Here, an exceptional result was that CD11b^+^Ly-6C^hi^ monocytes in BM showed no altered phenotypic levels by depletion of CD11c^hi^ DCs. This finding indicates that, due to the spatial effect of colocalization in the marginal zone^43^, CD11c^hi^ DCs in the spleen may regulate the differentiation and function of splenic CD11b^+^Ly-6C^hi^ monocytes, which are subsequently mobilized into the CNS by viral infection. This notion is contrast with the recent results showing that the function of Ly-6C^hi^ monocytes is pre-emptively educated in BM prior to the arrival of monocytes at local inflammation site^33^. It is thought that this contrast result may be derived from different context of infection model and/or depletion methods. The CCL2/CCR2 axis is a very important player in the context of viral encephalitis, facilitating emigration of CD11b^+^Ly-6C^hi^ monocytes from BM into blood and entry into immune-privileged CNS^5^,^9^. The major producer of CCL2 appears to be different in the CNS, depending on pathogens, with microglia serving as an important source during HSV infection^44^, neurons in the case of WNV^5^, and astrocytes in HIV encephalitis^45^. Our results support the possibility that infiltrated CD11b^+^Ly-6C^hi^ monocytes may be one of several producers to recruit CD11b^+^Ly-6C^hi^ monocytes themselves. No matter the source of CCL2, disruption of the CCL2/CCR2 axis can significantly reduce the infiltration of CD11b^+^Ly-6C^hi^ monocytes into the brain^9^. Thus, another interesting finding was that CD11b^+^Ly-6C^hi^ monocytes generated in CD11c^hi^ DC-ablated environment showed faster and more skewed migration into inflamed CNS and blood. This altered migration of monocytes generated in DC-ablated mice can be explained by moderately enhanced expression of CCR2 molecules in CD11b^+^Ly-6C^hi^ monocytes infiltrated in brain. Similar to a previous report^9^, we found that CCR2 was not involved in the migration of CD11b^+^Ly-6C^hi^ monocytes from blood into the inflamed CNS because the same ratio of labeled CD11b^+^Ly-6C^hi^ monocytes from vehicle- and DT-treated CD11c-DTR mice was observed in both blood and CNS. Although CCR2 molecule is most important in CD11b^+^Ly-6C^hi^ monocyte trafficking in the brain, additional work will be needed to define whether there is also a reduction in T cell subsets and other leukocytes at a later stage.

However, interpretation of the data generated in this study requires caution because DCs are important player to initiate adaptive immunity against pathogens. Impaired adaptive immunity developed in the absence of DCs might be able to promote JE progression. Indeed, survived CD11c^hi^ DC-depleted mice at 7 dpi generated diminished humoral and T-cell responses specific for JEV antigen. Because CD11c-DTR mice infected with JEV usually exhibited neurological disorder at 3–4 dpi before fully functional CD4^+^ and CD8^+^ T cell responses are induced, rapid innate cell responses such as CD11b^+^Ly-6C^hi^ monocytes appear more critical to control JE progression at the early stage. In contrast, impaired humoral and T cell responses generated in the absence of CD11c^hi^PDCA-1^int/lo^ DCs are likely to be involved in the exacerbation of JE progression at later stage started from around 5 dpi. Furthermore, the role of CD4^+^Foxp3^+^ Tregs in acute viral diseases is still debatable^32^. Recent work implicates CD4^+^Foxp3^+^ Tregs in the control of neuroinflammation caused by WNV^32^, wherein peripheral expansion of Treg was associated with mild inflammation, but reduced Treg levels were associated with WNV encephalitis. Thus, the reduction of CD4^+^Foxp3^+^ Treg cells in DC-ablated mice appears failed to control neuroinflammation. Also, DC ablation triggers a progressive myeloproliferative disorder in situations of constitutive DC ablation using cell-type specific expression of a suicide gene^31^. Moreover, the proliferative responses of HSCs in DC-deficient mice have been observed in several pathologic diseases such as sepsis, which induces a transient reduction of the DC population^46^. Similarly, a transient reduction of DC subsets was also observed in JEV infection^47^, which could alter hematopoietic cell responses. Here, CD11c^hi^ DC-depleted mice did not show any apparent increases in numbers of myeloid-derived cells in the spleen before viral infection, except for a moderate increase in frequency of CD11b^+^Ly-6C^hi^ cells. However, DC-ablated mice induced significantly increased frequency and absolute number of splenic CD11b^+^Ly-6C^hi^ monocytes during JE progression (data not shown). This observation describes a critical role for CD11c^hi^ DCs in the feedback regulation of steady- as well as pathologic-state hematopoiesis, which may be mediated by growth factors such as Flt3-L and GM-CSF in the absence of DCs. Therefore, our current findings suggest that peripheral CD11c^hi^ DCs play a critical and unexpected role in preserving the immune-privileged CNS *via* regulating the differentiation, function, and trafficking of CD11b^+^Ly-6C^hi^ monocytes derived by hematopoietic stem cells.

The debatable role of CD11b^+^Ly-6C^hi^ monocytes in the course of neuroinflammation caused by pathogenic CD4^+^ T cells or neurotropic virus initiates a new era to their differentiation lineage and immunopathologic role in immune-privileged CNS. On the basis of tight interactions of DCs in the monocyte-phagocyte differentiation system, it is presumable that selective depletion of specific DC subsets changes the features of monocyte subpopulations and that DCs contribute to the maintenance of a disciplined monocytic system to preserve the immune-privileged CNS. Although the precise molecular mechanisms and specific subpopulations of CD11c^hi^ DCs involved in their control of monocyte differentiation to foster immune privilege of the CNS remains to be defined, our results provide new insight to a critical and unrecognized role of peripheral CD11c^hi^ DCs in the control of CNS inflammation through regulating the differentiation, function, and trafficking of CD11b^+^Ly-6C^hi^ monocytes.

## Methods

### Ethics statement

All animal experiments described in the present study were conducted at Chonbuk National University according to the guidelines set by the Institutional Animal Care and Use Committees (IACUC) of Chonbuk National University (http://lac.honamlife.com/research/research_05.php) and were pre-approved by the Ethical Committee for Animal Experiments of Chonbuk National University (Permission code 2013–0028). Animal research protocol in this study followed the guideline set up by the nationally recognized Korea Association for Laboratory Animal Sciences (KALAS). All experimental protocols requiring biosafety were approved by Institutional Biosafety Committees (IBC) of Chonbuk National University.

### Animals

C57BL/6 (H-2^b^) mice (4–6 weeks old) were purchased from Samtako (O-San, Korea). CD11c-DTR transgenic (Tg) mice (B6.FVB-Tg Itgax-DTR/EGFP 57Lan/J [DTR]) and CCR2 knockout (KO) mice were obtained from Jackson Laboratories (Bar Harbor, ME, USA), and CD11c-DTR·CCR2 KO mice were generated by crossing CD11c-DTR Tg mice with CCR2 KO mice. All mice were genotyped and bred in the animal facilities of Chonbuk National University.

### Cells, viruses, antibodies, and reagents

The JEV Beijing-1 strain was propagated in a mosquito cell line (C6/36) using DMEM supplemented with 2% FBS, penicillin (100 U/ml), and streptomycin (100 U/ml)^47^. The virus stocks were titrated by conventional plaque assay using BHK-21 cells (CCL-10; American Type Culture Collection), and stored in aliquots at −80 °C until use. The mAbs used for the flow cytometric analysis and other experiments were obtained from eBioscience (San Diego, CA, USA) or BD Biosciences (San Diego, CA, USA) (Table S1). MHC II (I-A^b^)-restricted epitope peptide (OVA_323−339_) of chicken ovalbumin and JEV epitope peptides (NS1_132−145_ and NS3_563−574_ for CD4^+^ T cells and NS4B_215−223_ for CD8^+^ T cells) were chemically synthesized at Peptron Inc. (Daejeon, Korea). Diphtheria toxin (DT) was purchased from Sigma-Aldrich (St. Louis, MO, USA). The primers specific for JEV and cytokines (Table S2) were synthesized at Bioneer Corp. (Daejeon, Korea) and were used for PCR amplification of target genes.

### Quantification of viral burden and cytokine expression

Viral burden and expression of cytokines (IL-1β, IL-6, IL-10, IL-12, TGF-β, and TNF-α), and chemokines (CCL2, CCL3, CCL4, CCL5, CXCL2, and CXCL10) in inflammatory and lymphoid tissues were determined by quantitative SYBR Green-based real-time RT-PCR (real-time qRT-PCR). Mice were infected intraperitoneally (i.p.) with JEV (1.5 × 10^7^ PFU) and tissues including brain, spinal cord, and spleen were harvested at 2, 4, and 6 dpi. Total RNAs extracted from tissues using easyBLUE (iNtRON, INC., Daejeon, Korea) were employed in real-time qRT-PCR using a CFX96^TM^ Real-Time PCR Detection system (Bio-Rad Laboratories, Hercules, CA, USA). Following reverse transcription of total RNAs with High-Capacity cDNA Reverse Transcription Kits (Applied Biosystems, Foster City, CA, USA), the reaction mixture contained 2 μl of template cDNA, 10 μl of 2× SYBR Primix Ex Taq, and 200 nM primers in a final volume of 20 μl. The reactions were denatured at 95 °C for 30 s and then subjected to 45 cycles of 95 °C for 5 s, and 60 °C for 20 s. After the reaction cycle was completed the temperature was increased from 65 °C to 95 °C at a rate of 0.2 °C/15 s, and the fluorescence was measured every 5 s to construct a melting curve. A control sample that contained no template DNA was run with each assay, and all determinations were performed at least in duplicate to ensure reproducibility. The authenticity of the amplified product was determined by melting curve analysis. Viral RNA burden in the infected samples was expressed as viral RNA copies per microgram of RNA, and the relative ratios of cytokines and chemokines in infected samples to uninfected samples were determined. All data were analyzed using the Bio-Rad CFX Manager, version 2.1 analysis software (Bio-Rad Laboratories).

### ELISA for cytokines, chemokines, and hematopoietic factors

Sandwich ELISA was used to determine the serum levels of IL-6 and TNF-α cytokines. Flt3-L level was determined by commercialized ELISA kit (R&D Systems, Minneapolis, MN, USA). GM-CSF, CCL2, and CXCL1 were measured by cytokine bead array (CBA) technique (eBioscience), according to the manufacturer’s protocol.

### Leukocyte isolation and CD11b^+^Ly-6C^hi^ sorting from the CNS

Mice infected with JEV were perfused with 30 ml of HBSS containing heparin on day 3 or 5 pi *via* cardiac puncture of the left ventricle. Brains were then harvested and homogenized by gently pressing them through a 100-mesh tissue sieve and digested with 25 μg/ml of collagenase type IV (Worthington Biochem, Freehold, NJ, USA), 0.1 μg/ml trypsin inhibitor *Nα*-*p*-tosyl-L-lysine chloromethyl ketone, 10 μg/ml DNase I (Amresco, Solon, OH, USA), and 10 mM HEPE in HBSS for 1 h at 37 °C with shaking. Cells were separated by using Optiprep density gradient (18/10/5%) centrifugation at 800 × g for 30 min (Axis-Shield, Oslo, Norway), after which the cells were collected from the 18% to 10% interface and washed twice with PBS. Cells were then counted and stained for CD11b, Gr-1, Ly-6G, Ly-6C, CD3ε, CD4, and CD8α with directly conjugated antibodies (eBioscience) for 30 min at 4 °C. Finally, the cells were fixed with 10% buffered formaldehyde. Data collection and analysis were performed with FACSCalibur flow cytometer (Becton Dickson Medical Systems, Sharon, MA, USA) and FlowJo (ver. 7.6.5; Tree Star, San Carlos, CA, USA) software. In some experiments, CD11b^+^Ly-6C^hi^ monocytes were purified from brain leukocytes and splenocytes by applying cells to a FACS Aria sorter (Becton Dickson Medical Systems). Purified CD11b^+^Ly-6C^hi^ monocytes showed >95% purity.

### Cytospin, histology, and confocal microscopy

To cytospin CD11b^+^Ly-6C^hi^ and CD11b^+^Ly-6G^hi^ cells on Cytoslide (Thermo Scientific, Asheville, NC, USA), sorted cells were centrifuged at 1,500 rpm for 10 min using a CytoSpin 4 Cytocentrifuge (Thermo Scientific), and were then stained with hematoxylin and eosin (H&E). For histological examination of the brain, brain derived from mock and JEV-infected mice were embedded in paraffin and 10-μm sections were prepared and stained with H&E by the Pathology Lab (College of Veterinary Medicine, Chonbuk National University, Jeonju, Korea). Sections were analyzed using a Nikon Eclipse E600 microscope (Nikon, Tokyo, Japan). For confocal microscopy of brain tissue, brains were collected and frozen in optimum cutting temperature (OCT) compound (Sakura Finetechnical Co., Tokyo, Japan) following vigorous heart perfusion with HBSS. Sections of 6–7 μm thick were cut, air-dried, and fixed in cold solution (1:1 mixture of acetone and methanol) for 15 min at −20 °C. Non-specific binding was blocked with 10% normal goat serum and cells were permeabilized with 0.1% Triton X-100. Staining was performed by incubating sections overnight in a moist chamber at 4 °C with antibodies to JEV (Abcam, Cambridge, UK), MAP-2 (microtubule-associated protein 2; Millipore, Billerica, MA, USA), and GFAP (glial fibrillary acidic protein; Dako, Glostrup, Denmark), as well as biotin-conjugated anti-mouse CD11b (BD Biosciences) antibody. Primary antibodies were detected with secondary FITC-conjugated goat anti-mouse IgG, FITC-conjugated streptavidin, and PE-conjugated goat anti-mouse Ab. Nuclei were counterstained with DAPI (4'6-diamidino-2-phenylindole) (Sigma-Aldrich). Finally, the fluorescence was observed by confocal laser scanning microscope (Carl Zeiss, Zena, Germany).

### Proliferation analysis of CD11b^+^Ly-6C^hi^ monocytes

Proliferation of CD11b^+^Ly-6C^hi^ monocytes in the splenocytes incubated with sera was assessed by incorporation of 5-ethynyl-2’-deoxyuridine (EdU) using the Click-iT EdU imaging kit (Invitrogen, Carlsbad, CA, USA), according to the manufacturer’s protocol. Proliferated monocytes were determined by flow cytometric analysis after gated on CD11b^+^Ly-6C^hi^ monocytes.

### Generation of BM chimeric mice

C57BL/6 mice (6–7 weeks old) protected by head shielding were γ-irradiated with one dose of 950 rads. Within 12 h, mice were reconstituted with 10^7^ donor BM cells derived from CD11c-DTR, CCR2 KO, or CD11c-DTR·CCR2 KO mice. The recipient mice were given sulfamethoxazole and trimethoprim in drinking water for 10 days after irradiation. 4–6 weeks after irradiation, mice were infected with JEV after adoptive transfer of CD11b^+^Ly-6C^hi^ monocytes (1.5 × 10^6^ cells/mouse).

### Trafficking analysis of sorted CD11b^+^Ly-6C^hi^ monocytes from DT-treated CD11c-DTR mice in the CNS and lymphoid tissues

Differential labeling of CD11b^+^Ly-6C^hi^ monocytes was used to evaluate trafficking of sorted CD11b^+^Ly-6C^hi^ monocytes from vehicle- or DT-treated CD11c-DTR mice into the CNS and lymphoid tissues. After injection of DT or vehicle 2 times at a 1-day interval, CD11b^+^Ly-6C^hi^ monocytes were purified by FACS Aria sorter following enrichment of CD11b^+^ cells by PE-labeled MACS beads (Miltenyi Biotec GmbH, Bergisch Gladbach, Germany). CD11b^+^Ly-6C^hi^ monocytes were resuspended at 10^7^ cells/ml in RPMI 1640 complete medium (containing 10% FBS, 1% L-glutamine, 1% nonessential amino acids, and 1% penicillin/streptomycin). CMFDA Cell Tracker Green (Invitrogen) for sorted CD11b^+^Ly-6C^hi^ monocytes from vehicle-treated CD11c-DTR mice (Veh^CMFDA^) or CMTMR Cell Tracker Orange (Invitrogen) for sorted CD11b^+^Ly-6C^hi^ monocytes from DT-treated CD11c-DTR mice (DT^CMTMR^) were added for final concentrations of 5 μM and 10 μM, respectively, and incubated in a water bath at 37^o^C for 10 min. Cells were washed three times with cold RPMI 1640 complete medium and resuspended in sterile PBS at a 1:1 ratio (Input cell). A total of 5.0 × 10^6^ CD11b^+^Ly-6C^hi^ monocytes (2 doses, each 2.5 × 10^6^ cells) were injected intravenously into JEV-infected CCR2 KO mice at 4 dpi. Brain, BM, and spleen tissues as well as blood were harvested 20 h after injection of donor cells and the infiltrated cells were analyzed for the presence of labeled cells by FACS. To analyze brain leukocytes, vigorous heart perfusion was performed before harvesting brain tissue. Cell distribution of labeled donor cells was calculated based on the ratio detected in the output organ (CNS, blood, BM, and spleen) compared with the initial input ratio of labeled cells at the time of cell transfer (DT^CMTMR^:Veh^CMFDA^).

### JEV-specific antibody and CD4^+^/CD8^+^ T-cell responses

JEV-specific IgM level in sera of survived CD11c-DTR mice following JEV infection were determined by conventional ELISA using JEV E glycoprotein antigen (Abcam, Cambridge, UK) at 7 dpi. JEV-specific CD4^+^ and CD8^+^ T cell responses were determined by intracellular CD154^48^,^49^ and IFN-γ staining in response to stimulation with JEV epitope peptides, respectively. CD11c-DTR mice given vehicle or DT were infected i.p. with JEV (1.5 × 10^7^ PFU/mouse) and survived mice were sacrificed at day 7 pi and splenocytes were prepared. The erythrocytes were depleted by treating single-cell suspension with ammonium chloride-containing Tris buffer (NH_4_Cl-Tris) for 5 min at 37^o^C. The splenocytes were cultured in 96-well culture plates (5 × 10^5^ cells/well) in the presence of synthetic epitope peptides (12 h-incubation for CD4^+^ T cells in the presence of PE-CD154 antibody and 8 h-incubation for CD8^+^ T cells). Monensin at the concentration of 2 μM was added to antigen-stimulated cells 6 h before harvest. The cells were washed twice with PBS, and surface stained for FITC-anti-CD4 or CD8 antibodies for 30 min at 4^o^C. After fixation, the cells were washed twice with permeabilization buffer (eBioscience) and then stained with PE-anti-IFN-γ antibody in permeabilization buffer for CD8^+^ T cells. Finally, the cells were washed twice with PBS and fixed using fixation buffer. Sample analysis was performed with FACS Calibur flow cytometer (Becton Dickson Medical Systems) and FlowJo software (ver. 7.6.5; Tree Star).

### Statistical analysis

All data were expressed as the average ± standard deviation, and statistically significant differences between groups were analyzed by unpaired two-tailed Student’s *t*-test for leukocyte population analysis and *in vitro* experiments or ANOVA and post-hoc testing for multiple comparisons of the mean. The statistical significance of viral burden and *in vivo* cytokine gene expression were evaluated by Mann-Whitney test or unpaired two-tailed Student’s t-test. Kaplan-Meier survival curves were analyzed by the log rank test. A *p*-value ≤ 0.05 was considered significant. All data were analyzed using Prism software (GraphPadPrism4, San Diego, CA, USA).

## Additional Information

**How to cite this article**: Kim, J. H. *et al.* CD11c^hi^ Dendritic Cells Regulate Ly-6C^hi^ Monocyte Differentiation to Preserve Immune-privileged CNS in Lethal Neuroinflammation. *Sci. Rep.* **5**, 17548; doi: 10.1038/srep17548 (2015).

## Supplementary Material

Supplementary Information
