# Supplementary material for: CD11chi Dendritic Cells Regulate Ly-6Chi Monocyte Differentiation to Preserve Immune-privileged CNS in Lethal Neuroinflammation
Source: Sci Rep. 2015 Dec 2;5:17548. doi: 10.1038/srep17548 (PMC4667186; doi:10.1038/srep17548)
Supplement: Supplementary Information [file srep17548-s1.pdf]

CD11c^{hi} Dendritic Cells Regulate Ly-6C^{hi} Monocyte Differentiation to Preserve Immune-privileged CNS in Lethal Neuroinflammation

Jin Hyoung Kim[1], Jin Young Choi[1], Seong Bum Kim[1], Erdenebelig Uyangaa[1], Ajit Mahadev Patil[1], Young Woo Han[1], Sang-Youel Park[1,2], John Hwa Lee[1,2], Koanhoi Kim[3] & Seong Kug Eo[1,2*]

[1]College of Veterinary Medicine and Bio-Safety Research Institute, Chonbuk National University, Iksan 54596, Republic of Korea

[2]Department of Bioactive Material Sciences, Graduate School, Chonbuk National University, Jeonju 54896, Republic of Korea

[3]Department of Pharmacology, Pusan National University, School of Medicine, Yangsan 50612, Republic of Korea

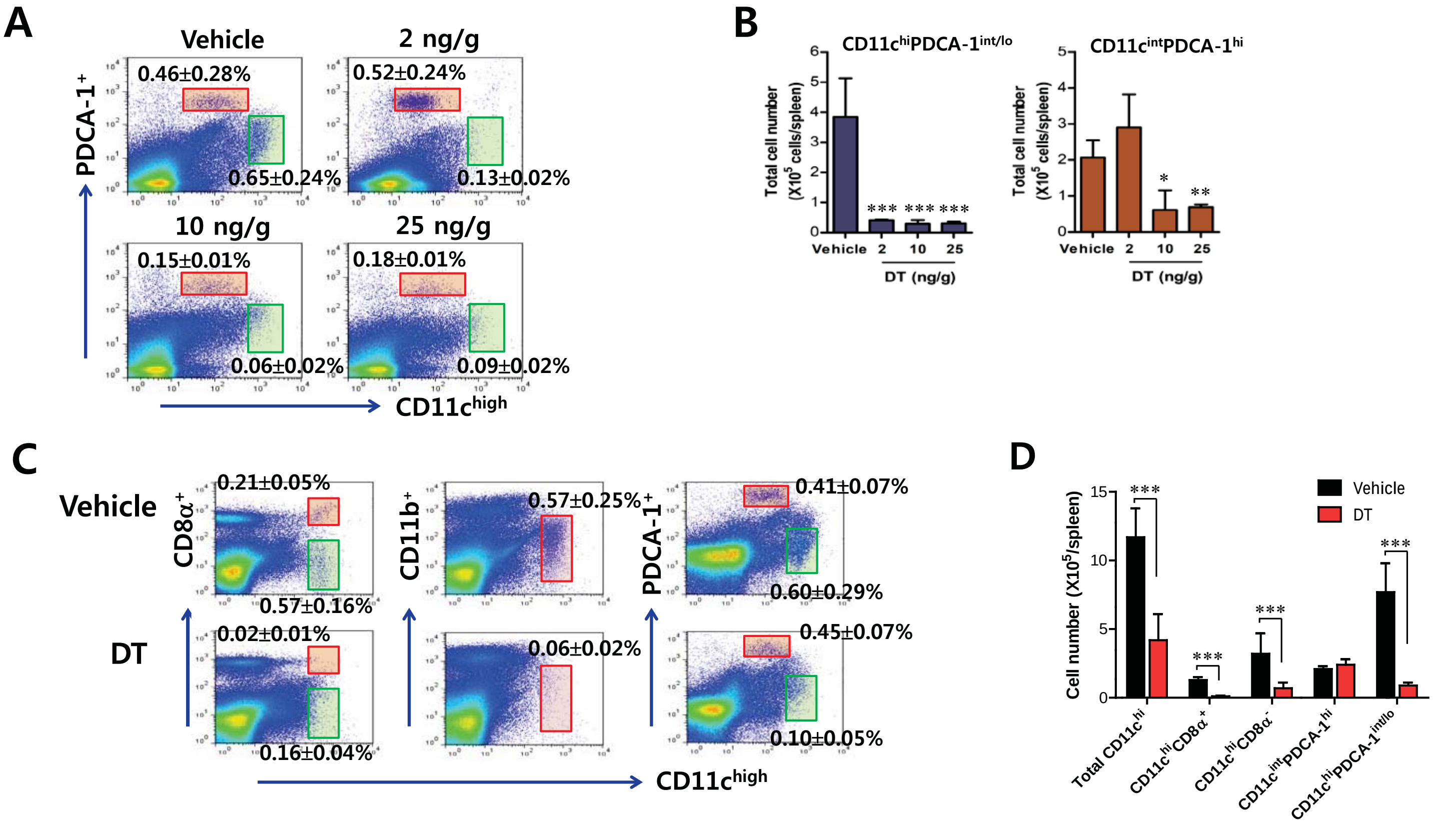

Figure S1. Selective ablation of CD11c^{hi}PDCA-1$^{int/lo}$ DCs by injection of low dose DT in CD11c-DTR mice. (A and B) Selective ablation of CD11c^{hi}PDCA-1$^{int/lo}$ DC by injection of low dose DT. The frequency (A) and number (B) of DC subsets (CD11c^{hi}PDCA-1$^{int/lo}$ and CD11c^{int}PDCA-1^{hi}) were determined by flow cytometric analysis 1 day after injection of the indicated DT doses (2, 10, and 25 ng/g) 2 times at one-day interval. (C and D) Distribution and absolute number of DC subpopulations after low dose DT injection. The frequency (C) and absolute number (D) of DC subpopulation (CD11c^{hi}CD8α^+, CD11c^{hi}CD11b$^+$, and CD11c^{int}PDCA-1^{hi}) were evaluated one day after injection of low DT dose (2 ng/g) two times at one-day interval. The values in representative dot-plot and graph denote the average ± SD of at least four mice per group. *, p<0.05; **, p<0.01; ***, p<0.001 compared with the levels of the vehicle-treated group.

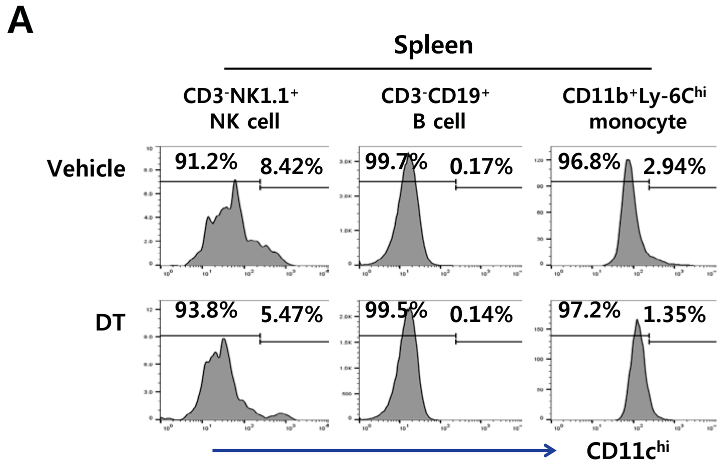

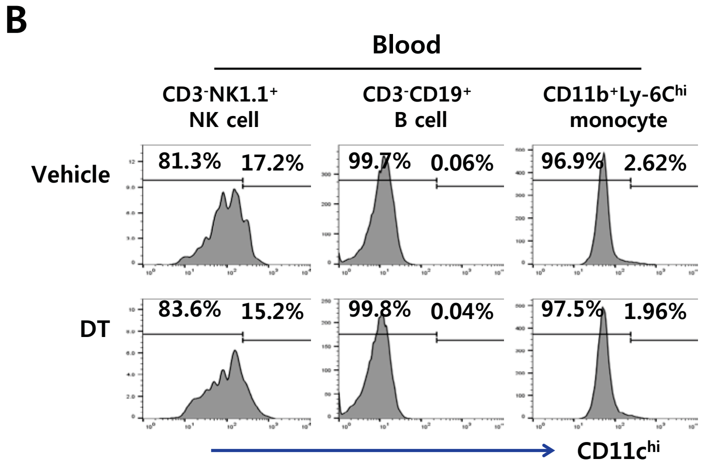

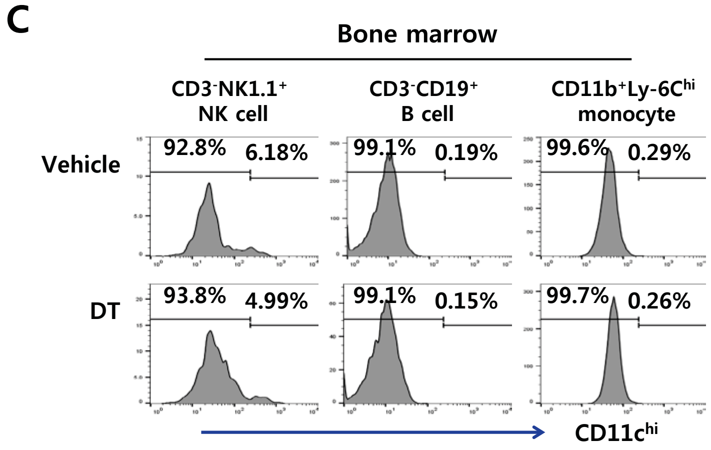

Figure S2. The effect of low dose DT injection on subpopulation of B, NK, and monocyte expressing CD11c molecule. The frequency of CD11c-expressing cells in NK cells ($CD3^-NK1.1^+$), B cells ($CD3^-CD19^+$) and monocytes ($CD11b^+Ly\text{-}6C^{hi}$) contained in spleen (A), blood (B), and bone marrow (C) of CD11c-DTR mice was monitored 1 day after injection of low dose DT (2 ng/g) 2 times at one-day interval. The values in representative histograms denote the average percentage of $CD11c^{hi}$ cells in the indicated cell population (*n*=4-5).

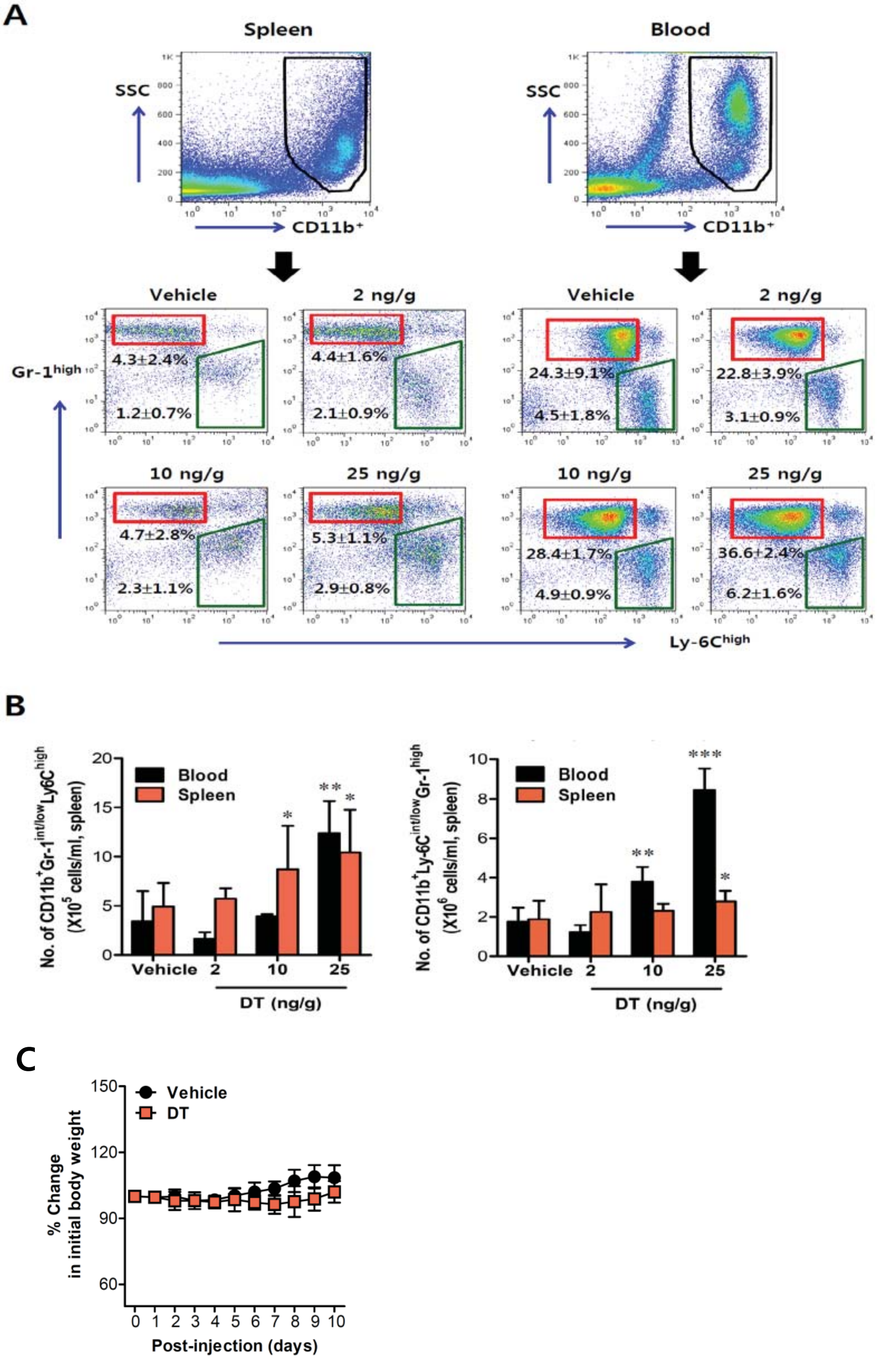

Figure S3. The injection of low dose DT in CD11c-DTR mice did not induce systemic neutrophilia as well as apparent changes in body weight. (A and B) The injection of low dose DT in CD11c-DTR mice did not induce systemic neutrophilia. The frequency (A) and number (B) of CD11b^{+}Ly-6C$^{int/lo}$Gr-1^{hi} and CD11c^{+}Ly-6C^{hi}Gr-1$^{int/lo}$ cells per milliliter of blood and in the spleen of CD11c-DTR mice were determined 1 day after injection of the indicated DT doses 2 times at one-day interval. The values in representative dot-plot and graphs denote the average ± SD of at least four mice per group. (C) Changes in body weight of CD11c-DTR mice injected with DT. CD11c-DTR mice (n=5) were injected i.p. with DT every other day for 7 days, and body weigh was examined daily. Data are expressed as the average percentage ± SD of body weight relative to initial body weight. *, p<0.05; **, p<0.01; ***, p<0.001 compared with vehicle group.

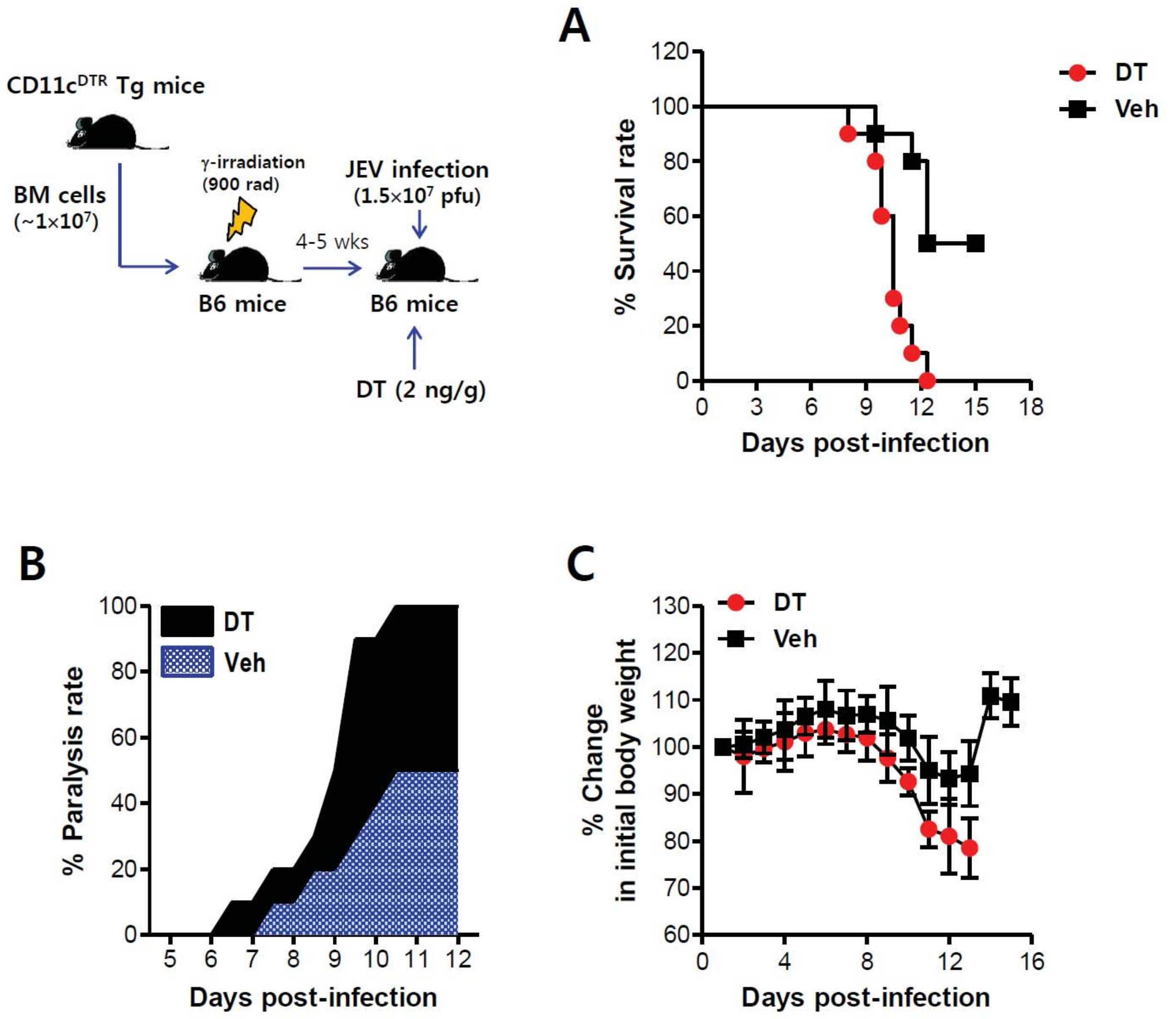

Figure S4. Susceptibility of CD11c-DTR BM-cell recipients to JE. BM cells isolated from CD11c-DTR were injected to γ-irradiated B6 recipients, which were subsequently infected with JEV (1.5×10^7 pfu/mouse) 4-5 weeks later. CD11c-DTR BM-cell recipients were injected i.p. with DT (2 ng/g) every other day from -1 to 7 days after JEV infection. (A) Survival rate. Surviving mice were monitored until day 15 after infection. (B) The proportion of mice showing paralysis from 4 to 12 days after infection. (C) Changes in body weight. The data is expressed as the average ± SD of weight relative to the time of challenge (*n*=10). The pictures of mice were drawn by S.K. Eo.

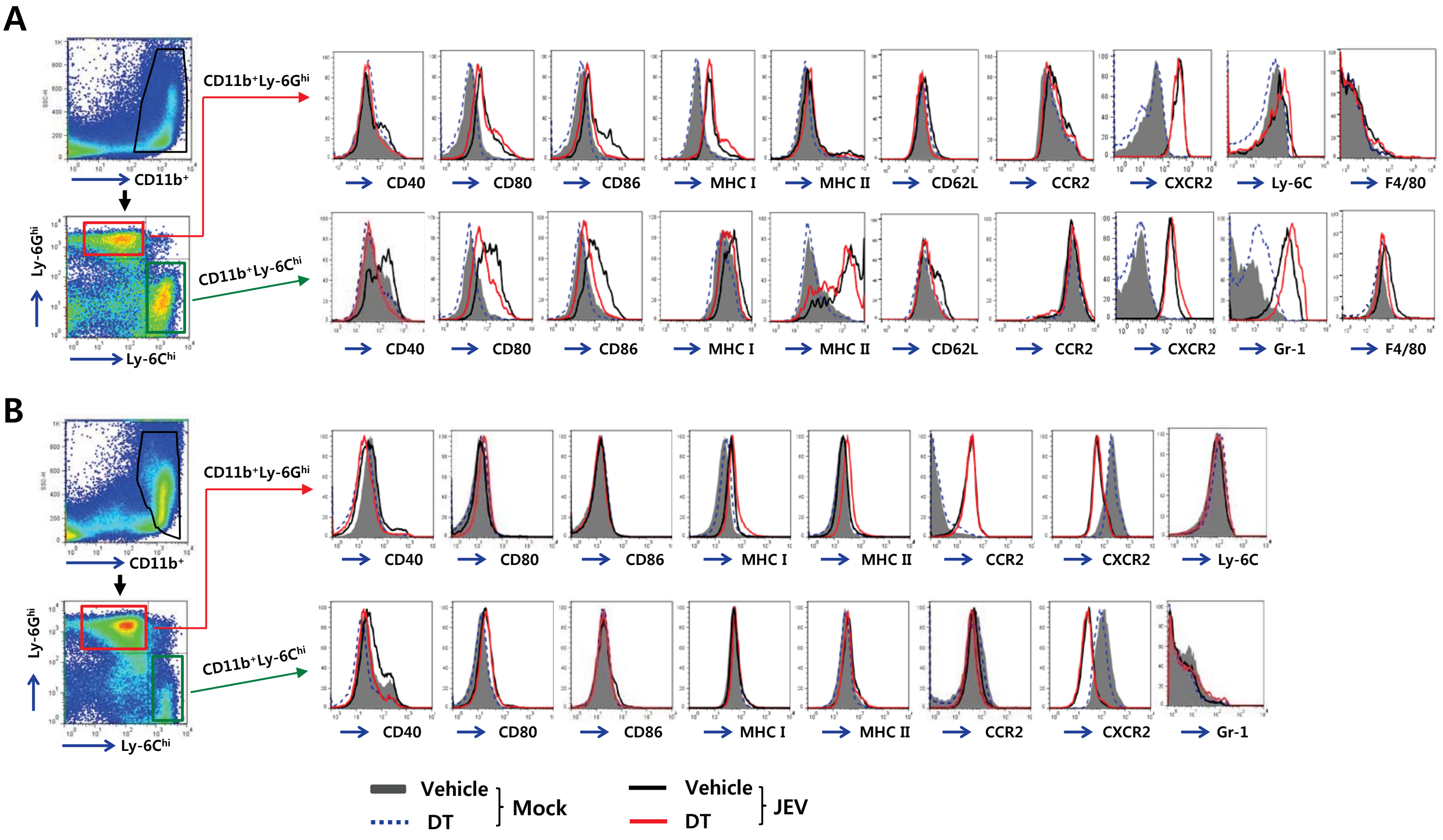

Figure S5. Differential activation levels of CD11b$^+$Ly-6C^{hi} monocytes in the spleen and bone marrow of CD11c^{hi}PDCA-1$^{int/lo}$ DCs-depleted mice. CD11c-DTR mice were injected i.p. with DT (2 ng/g) every other day from -1 to 7 days after JEV infection (1.5×10^7 pfu/mouse). The differentiation levels of CD11b$^+$Ly-6C^{hi} monocytes and CD11b$^+$Ly-6G^{hi} granulocytes in the spleen (A) and bone marrow (B) were analyzed by flow cytometric analysis at 3 dpi. The histograms are representative of three individual experiments (*n*=5-7).

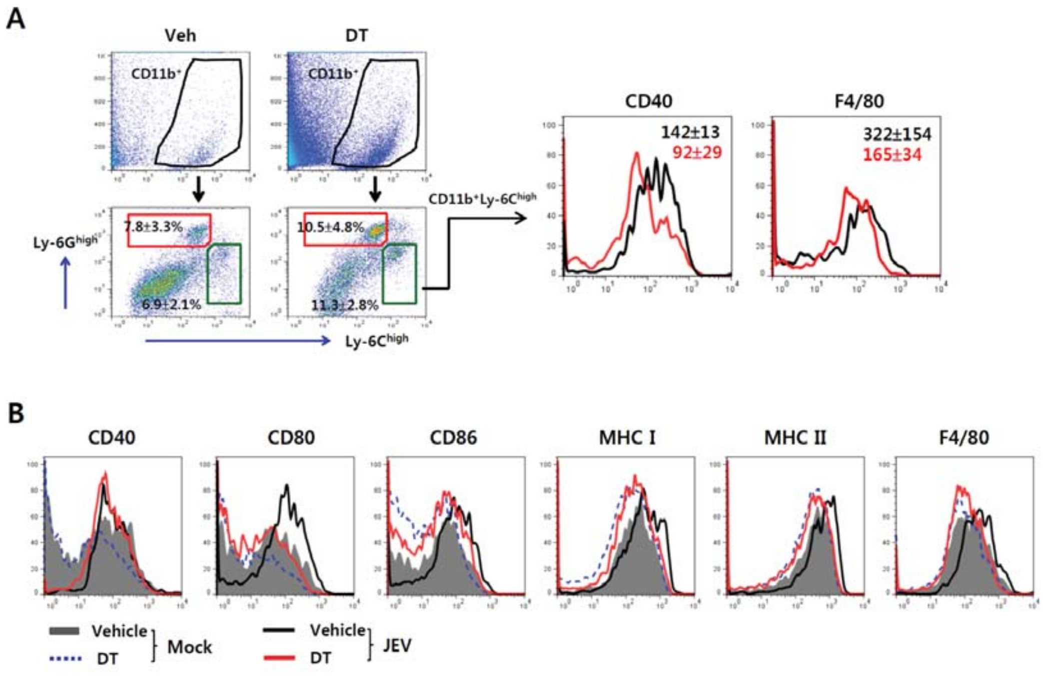

Figure S6. Differentiation levels of CD11b+Ly-6G$^{int/lo}$Ly-6C^{hi} monocytes in CD11c^{hi}PDCA-1$^{int/lo}$ DC-depleted recipients of CD11c-DTR BM cells. BM cells isolated from CD11c-DTR were injected to γ-irradiated B6 recipients, which were infected with JEV (1.5×10^7 pfu/mouse) 4-5 weeks later. CD11c-DTR BM-cell recipients were injected i.p. with DT (2 ng/g) every other day from -1 to 7 dpi. (A) Differentiation levels of CD11b$^+$Ly-6G$^{int/lo}$Ly-6C^{hi} monocytes infiltrated into the CNS. After vigorous heart perfusion at 3rd dpi, the levels of differentiation marker CD40 and F4/80 in CNS-infiltrated CD11b$^+$Ly-6G$^{int/lo}$Ly-6C^{hi} monocytes were determined by flow cytometric analysis. (B) Differentiation levels of CD11b$^+$Ly-6G$^{int/lo}$Ly-6C^{hi} monocytes in the spleen. The differentiation levels of CD11b$^+$Ly-6G$^{int/lo}$Ly-6C^{hi} monocytes in the spleen were analyzed by flow cytometric analysis 3 dpi. The histograms are representative of three individual experiments (*n*=3).

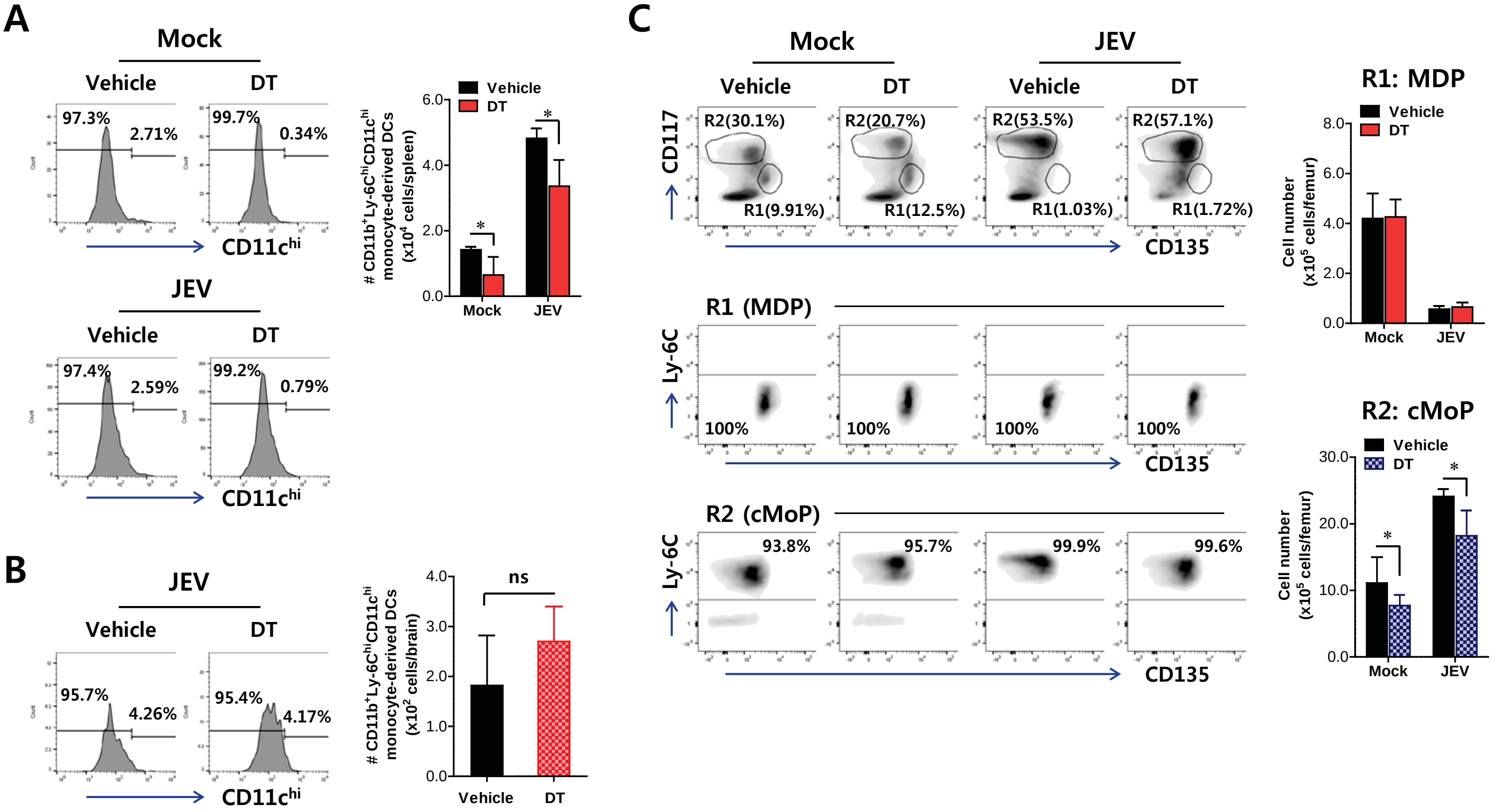

Figure S7. The number of CD11b$^+$Ly-6C^{hi} monocyte-derived DCs and MDP/cMoP in CD11c-DTR mice. CD11c-DTR mice were injected i.p. with DT every other day from -1 to 7 days after JEV infection (1.5×10^7 pfu/mouse). (A and B) The frequency and number of CD11b$^+$Ly-6C^{hi} monocyte-derived DCs. CD11b$^+$Ly-6C^{hi} monocyte-derived DCs were enumerated in the spleen (A) and brain (B) by flow cytometric analysis 3 dpi. Left histograms are representative of at least four individual experiments (n=3-4) after gating on CD11b$^+$Ly-6C^{hi} monocytes; Right graphs denote the average ± SD. (C) The frequency and number of MDP and cMoP in BM of CD11c-DTR mice. The frequency and number of MDP (R1:CD117$^+$CD115$^+$CD135$^+$Ly-6C$^-$CD11b$^-$Lin$^-$) and cMoP (R2: CD117$^+$CD115$^+$CD135$^-$Ly-6C$^+$CD11b$^-$Lin$^-$) in BM of CD11c-DTR mice were determined by flow cytometric analysis 3 dpi. Top dot-plots show cell populations after gating on CD115$^+$CD11b$^-$ in Lin$^-$ (CD3$^-$CD19$^-$NK1.1$^-$Ly-6G$^-$); middle and bottom dot plots represent R1(MDP) and R2(cMoP), respectively. Right graphs shows the average ± SD of total MDP and cMoP numbers contained in one femur of CD11c-DTR mice. *p<0.05 compared with the indicated groups; ns, no statistical significance.

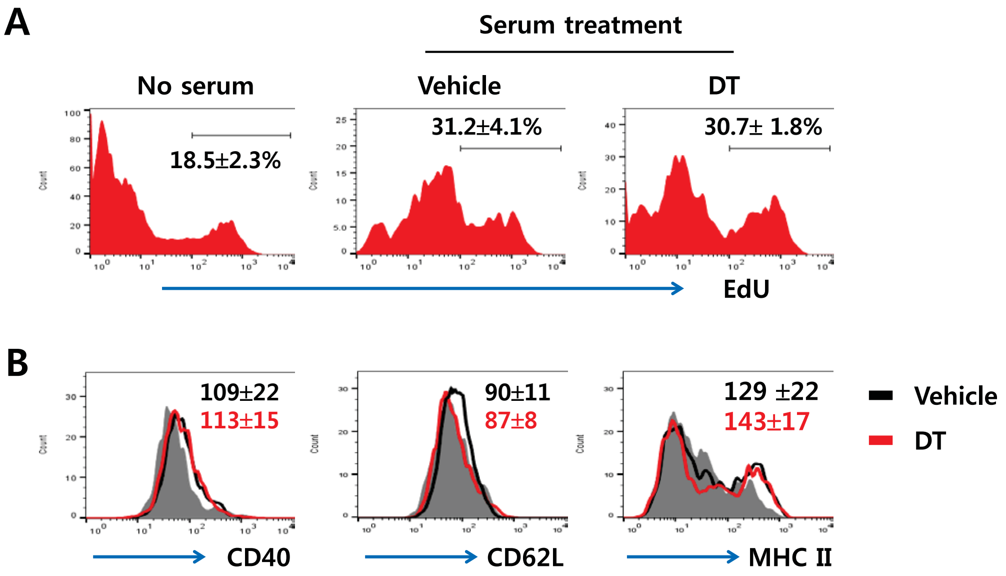

Figure S8. The proliferation and phenotypic changes of CD11b⁺Ly-6Cʰⁱ monocytes by sera derived from DT-treated CD11c-DTR mice that were not infected with JEV. (A) Proliferation of CD11b⁺Ly-6Cʰⁱ monocytes. The splenocytes prepared from DC-depleted mice were incubated with sera that were obtained from uninfected CD11c-DTR mice. The proliferation of CD11b⁺Ly-6Cʰⁱ monocytes was assessed by EdU incorporation after a 48-h incubation. The values in the representative histogram denote the average ± SD of % EdU-positive cells after gated on CD11b⁺Ly-6Cʰⁱ monocytes in quadruplicate wells. (B) Phenotypic changes in CD11b⁺Ly-6Cʰⁱ monocytes. After a 48-h incubation of DC-depleted splenocytes with sera derived from uninfected CD11c-DTR mice, the expression of phenotypic markers was determined by flow cytometric analysis. The values in the representative histogram denote the average ± SD of MFI of CD11b⁺Ly-6Cʰⁱ monocytes in quadruplicate wells. The gray line represents the expression levels of phenotypic markers in CD11b⁺Ly-6Cʰⁱ monocytes that were not treated with sera.

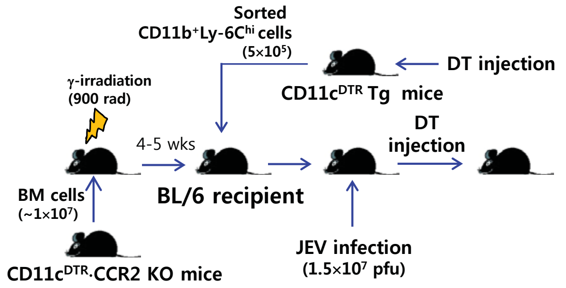

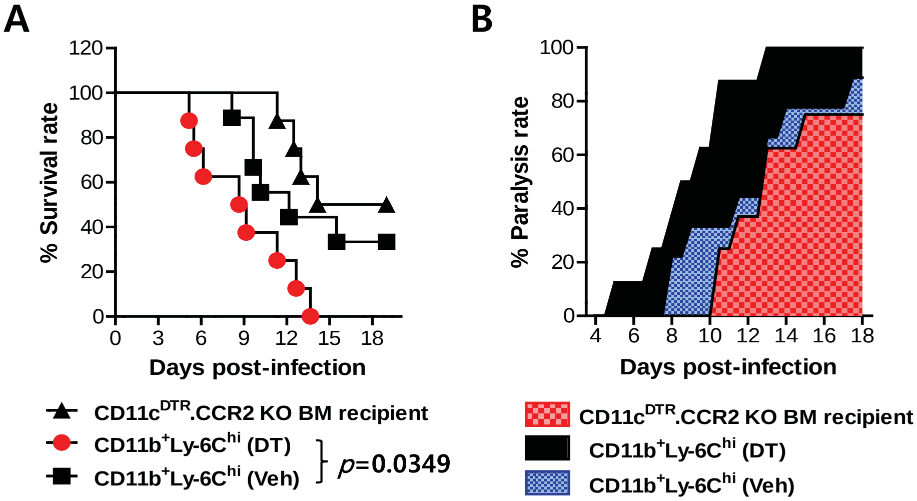

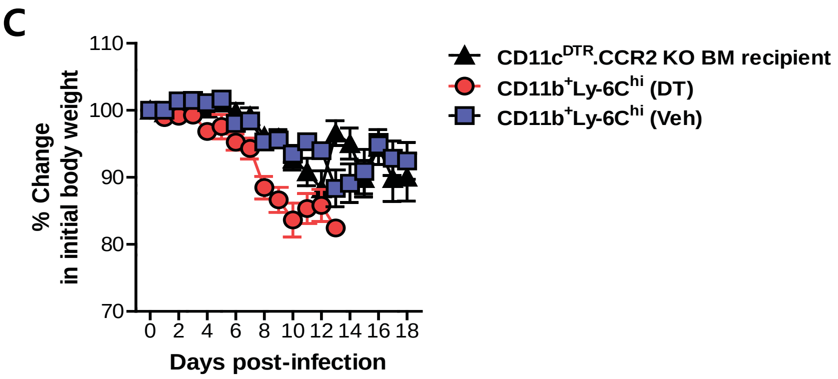

Figure S9. Proof that CD11b⁺Ly-6C^{hi} monocytes generated in CD11c^{hi}PDCA-1$^{int/lo}$ DC–depleted mice exacerbate JE without potential recovery of immature phenotypes. BM cells isolated from CD11c-DTR·CCR2 KO mice were injected into γ-irradiated B6 recipients, which then received adoptive transfer of CD11b⁺Ly-6C^{hi} monocytes (1.5×10^6 cells/mouse) sorted from DT- or vehicle-injected CD11c-DTR mice. CD11c-DTR·CCR2 KO BM recipients were injected i.p. with DT (2 ng/ml) every other day from -1 to 7 days after JEV infection. (A) Survival rates. The surviving proportion of CD11c-DTR·CCR2 KO BM recipients was monitored until day 20 after infection. (B) The proportion of CD11c-DTR·CCR2 KO BM recipients with paralysis. The proportion of the recipients showing paralysis was examined every 6 h from 4 dpi to 18 dpi. (C) Changes in body weight. Data is expressed as the average percentage ± SD of weight relative to the time of challenge (n=8-9). The pictures of mice were drawn by S.K. Eo.

Table S1. Antibodies used in flow cytometry and other experiments of this study

**Molecule**	**Clone**
CD3ε	RMA4-5
CD4	RM4-5
CD8α	53-6.7
CD11c	N418
CD11b	M1/70
CD40	HM40-3
CD40L(CD154)	MR1
CD44	IM7
CD45	30-F11
CD62L	MEL-14
CD69	H1.2F3
CD80	16-10A1
CD86	GL1
CD115	AFS98
CD117	2B8
CD135	A2F10
CCR2	475301
CXCR2	242216
MHC I (H-2b)	28-14-8
MHC II (I-Ab)	M5/114.15.2
F4/80	BM8
Gr-1	RB6-8C5
Ly-6G	1A8
Ly-6C	HK1.4
PDCA-1	eBio0927
IL-6	MP5-20F3
	MP5-32C11
IFN-γ	XMG1.2
TNF-α	1F3F3D4
	Polyclonal

Table S2. Specific primers used for determining the expression of cytokines, chemokines, and JEV RNA in real-time qRT-PCR.

Gene name[a]	Primer sequence (5'-3')[b]	Position cDNA	Gene Bank ID
IL-1β	FP: AAGTGATATTCTCCATGAGCTTTGT	535-559	NM_008361
	RP: TTCTTCTTTGGGTATTGCTTGG	679-700	
IL-6	FP: TGG GAA ATC GTG GAA ATG AG	209-228	NM_031168
	RP: CTC TGA AGG ACT CTG GCT TTG	442-462	
IL-10	FP: CAA CAT ACT GCT AAC CGA CTC CT	253-275	NM_010548
	RP: TGA GGG TCT TCA GCT TCT CAC	405-425	
IL-12p40	FP: GGA AGC ACG GCA GCA GAA TA	792-811	NM_008352
	RP: AAC TTG AGG GAG AAG TAG GAA TGG	948-971	
TGF-β	FP: GTG TGG AGC AAC ATG TGG AAC TCT	1355-1378	NM_011577
	RP: TTG GTT CAG CCA CTG CCG TA	1478-1497	
TNF-α	FP: CGT CGT AGC AAA CCA CCA AG	438-457	NM_013693
	RP: TTG AAG AGA ACC TGG GAG TAG ACA	564-587	
IFN-α	FP: TGTCTGATGCAGCAGGTGG	367-385	NM_008334.3
	RP: AAGACAGGGCTCTCCAGAC	514-532	
IFN-β	FP: TCCAAGAAAGGACGAACATTCG	106-121	NM_010510
	RP: TGAGGACATCTCCCACGTCAA	399-419	
CCL2	FP: AAA AAC CTG GAT CGG AAC CAA	347-367	NM_011333
	RP: CGG GTC AAC TTC ACA TTC AAA G	426-447	
CCL3	FP: CCA AGT CTT CTC AGC GCC AT	158-177	NM_011337.2
	RP: GAA TCT TCC GGC TGT AGG AGA AG	206-228	
CCL4	FP: TTC TGT GCT CCA GGG TTC TC	128-147	NM_013652.2
	RP: GAG GAG GCC TCT CCT GAA GT	388-407	
CCL5	FP: CCC TCA CCA TCA TCC TCA CT	77-96	NM_013653.3
	RP: CTT CTT CTC TGG GTT GGC AC	275-294	
CXCL2	FP: ATC CAG AGC TTG AGT GTG ACG C	194-215	NM_009140.2
	RP: AAG GCA AAC TTT TTG ACC GC	264-283	
CXCL10	FP: AAG TGC TGC CGT CAT TTT CT	84-103	NM_021274.2
	RP: CAT TCT TTT TCA TCG TGG CA	264-283	
CX_3CL1	FP: GTG CTG ACC CGA AGG AGA AA	306-325	NM_009142.3
	RP: CAC CCG CTT CTC AAA CTT GC	387-406	
JEV	FP: GGC TTA GCG CTC ACA TCC A	4132–4150	AB920399.1
	RP: GCT GGC CAC CCT CTC TTC TT	4207–4226	
β-actin	FP: TGG AAT CCT GTG GCA TCC ATG AAA C	885-909	NM_007393.3
	RP: TAA AAC GCA GCT CAG TAA CAG TCC G	1209-1233	

[a] IL, interleukin; TNF-α, tumor necrosis factor-α; IFN, interferon

[b] FP, forward primer; RP, reverse primer
